# Follistatin-like 1 (FSTL1) interacts with Wnt ligands and Frizzled receptors to enhance Wnt/β-catenin signaling in obstructed kidneys *in vivo*

**DOI:** 10.1016/j.jbc.2022.102010

**Published:** 2022-05-04

**Authors:** Yu Zhang, Yang Wang, Guoxun Zheng, Yang Liu, Jinhong Li, Huihui Huang, Chunhua Xu, Yelin Zeng, Xiaoyi Zhang, Jinzhong Qin, Chunsun Dai, Harald O. Hambrock, Ursula Hartmann, Bo Feng, Kingston Kinglun Mak, Youhua Liu, Hui-Yao Lan, Yu Huang, Zhi-Hua Zheng, Yin Xia

**Affiliations:** 1 Faculty of Medicine, School of Biomedical Sciences, The Chinese University of Hong Kong, Hong Kong, China; 2 iHuman Institute, Shanghai Tech University, Shanghai, China; 3 Department of Nephrology, Center of Nephrology and Urology, The Seventh Affiliated Hospital, Sun Yat-sen University, Shenzhen, China; 4 Department of Medicine, Beth Israel Deaconess Medical Center, Harvard Medical School, Boston, Massachusetts, USA; 5 The Key Laboratory of Model Animal for Disease Study of Ministry of Education, Model Animal Research Center, Nanjing University, Nanjing, China; 6 Center for Kidney Disease, The Second Affiliated Hospital, Nanjing Medical University, Nanjing, China; 7 Center for Biochemistry, Faculty of Medicine, University of Cologne, Cologne, Germany; 8 Guangzhou Regenerative Medicine and Health Guangdong Laboratory (GRMH-GDL), Guangzhou, China; 9 Department of Pathology, University of Pittsburgh School of Medicine, Pittsburgh, Pennsylvania, USA; 10 Department of Medicine and Therapeutics, Li Ka Shing Institute of Health Sciences, The Chinese University of Hong Kong, Hong Kong, China; 11 Guangdong-Hong Kong Joint Laboratory for Immune and Genetic Kidney Disease, Guangdong Provincial People’s Hospital and Guangdong Academy of Medical Sciences, Guangzhou, and The Chinese University of Hong Kong, Hong Kong, China; 12 Department of Biomedical Sciences, City University of Hong Kong, Kowloon Tong, Hong Kong, China

**Keywords:** FSTL1, Wnt, FZD, β-catenin, kidney fibrosis, α-SMA, alpha-smooth muscle actin, AKI, acute kidney injury, BMP, bone morphogenetic protein, CaMKII, Ca^2+^/calmodulin–dependent protein kinase II, CKD, chronic kidney disease, DMEM, Dulbecco's modified Eagle's medium, EC, extracellular calcium–binding domain, FBS, fetal bovine serum, Fn, fibronectin, FS, follistatin, FSTL1, follistatin-like 1, FSTL3, follistatin-like 3, FZD, Frizzled, HA, hemagglutinin, HEK293T, human embryonic kidney 293T cell line, IMCD3, inner medullary collecting duct 3, IP, immunoprecipitation, JNK, c-Jun N-terminal kinase, LRP, low-density lipoprotein receptor–related protein, MESD, mesoderm development LRP chaperone, NRK-49F, normal rat kidney 49 fibroblast cell, PDGFRβ, platelet-derived growth factor receptor beta, SNAP-FZD4, SNAP-tagged FZD4, SPARC, secreted protein, acidic and rich in cysteine, TGF-β, transforming growth factor beta, UUO, unilateral ureteral obstruction, VWC, von Willebrand factor type C–like, YAP, Yes-associated protein

## Abstract

Follistatin (FS)-like 1 (FSTL1) is a member of the FS-SPARC (secreted protein, acidic and rich in cysteine) family of secreted and extracellular matrix proteins. The functions of FSTL1 have been studied in heart and lung injury as well as in wound healing; however, the role of FSTL1 in the kidney is largely unknown. Here, we show using single-cell RNA-Seq that Fstl1 was enriched in stromal cells in obstructed mouse kidneys. In addition, immunofluorescence demonstrated that FSTL1 expression was induced in fibroblasts during kidney fibrogenesis in mice and human patients. We demonstrate that FSTL1 overexpression increased renal fibrosis and activated the Wnt/β-catenin signaling pathway, known to promote kidney fibrosis, but not the transforming growth factor β (TGF-β), Notch, Hedgehog, or Yes-associated protein (YAP) signaling pathways in obstructed mouse kidneys, whereas inhibition of FSTL1 lowered Wnt/β-catenin signaling. Importantly, we show that FSTL1 interacted with Wnt ligands and the Frizzled (FZD) receptors but not the coreceptor lipoprotein receptor–related protein 6 (LRP6). Specifically, we found FSTL1 interacted with Wnt3a through its extracellular calcium–binding (EC) domain and von Willebrand factor type C–like (VWC) domain, and with FZD4 through its EC domain. Furthermore, we show that FSTL1 increased the association of Wnt3a with FZD4 and promoted Wnt/β-catenin signaling and fibrogenesis. The EC domain interacting with both Wnt3a and FZD4 also enhanced Wnt3a signaling. Therefore, we conclude that FSTL1 is a novel extracellular enhancer of the Wnt/β-catenin pathway.

Kidney fibrosis is the final common pathway of progressive chronic kidney diseases (CKDs) and is characterized by the increase of interstitial fibroblasts and myofibroblasts, and the excessive production and accumulation of extracellular matrix proteins, such as collagens and fibronectins (Fns), within the kidney (1, 2, 3, 4). Kidney injury activates several critical signaling pathways, including the transforming growth factor beta (TGF-β), Wnt, Notch, Hedgehog, and Yes-associated protein (YAP) pathways, which collectively promote renal fibrogenesis (3).

The Wnt family of ligands controls a variety of cellular activities. Dysregulation of Wnt signaling has been implicated in human diseases, including tissue fibrosis and tumorigenesis. Wnts signal through the Frizzled (FZD) family of proteins as receptors, and low-density lipoprotein receptor–related proteins 5 and 6 (LRP-5 and LRP-6) as coreceptors, to stabilize β-catenin, which translocates into the nuclei to stimulate the transcription of Wnt target genes (5, 6). Wnt/β-catenin signaling has been found to promote renal fibrosis by increasing fibroblast differentiation, proliferation, and activity (7, 8, 9, 10, 11, 12, 13, 14, 15).

Follistatin (FS)-like 1 (FSTL1) is a secreted glycoprotein, which was initially identified as a TGF-β–inducible gene (16). FSTL1 belongs to the FS-SPARC (secreted protein, acidic and rich in cysteine) family of proteins containing the characteristic tandem of both extracellular calcium–binding (EC) domain and FS-like domain. Previous studies including ours have shown that the two other members of this family FS and FS-like 3 (FSTL3) bind to activin, myostatin, and bone morphogenetic protein (BMP) (17, 18, 19, 20, 21, 22), but not TGFβ (23). FSTL1 was also shown to bind to TGF-β superfamily proteins (24, 25, 26, 27) although it has relatively low sequence homology with FS and FSTL3. In this line, FSTL1 was found to prevent the formation of lung atelectasis by inhibiting BMP signaling during embryonic lung development (26), while it drove bleomycin-induced pulmonary fibrogenesis by facilitating TGF-β signaling in fibroblasts (27). However, the observations that FSTL1 interacted with the TGF-β1–receptor complex and promoted the downstream Smad2/3 signaling (27) have been challenged by a few other studies, which failed to show the interaction of FSTL1 with TGF-β1 and the regulation of TGF-β1–Smad3 signaling by FSTL1 (28, 29, 30). In addition, FSTL1 has been found to regulate several other pathways including the Disco-interacting protein 2 homolog A (DIP2A), AKT, extracellular signal–regulated kinase (ERK), and AMP-activated protein kinase (AMPK) pathways in cell type–dependent and context-dependent manners (31, 32, 33, 34, 35).

The biological functions of FSTL1 have been studied in heart injury (28, 31, 32, 33, 36), lung development and fibrosis (26, 27), arthritis (37, 38, 39), and wound healing (40, 41). In contrast to a profibrotic role of FSTL1 in the lung (27), heart (33), and liver (42), two previous studies on the kidney implicated protective and antifibrotic roles of FSTL1 in kidney injury and fibrosis (43, 44). In the present study, we found that FSTL1 promoted kidney fibrosis. Strikingly, this profibrotic activity was associated with the Wnt but not the TGF-β1 pathway. FSTL1 bound to Wnt ligands and FZD receptors, whereby it increased the presentation of Wnt ligands to FZD receptors to enhance Wnt/β-catenin signaling.

## Results

### FSTL1 was expressed in fibroblasts in fibrogenic kidneys

We first measured Fstl1 mRNA levels in different organs of normal adult mice. Fstl1 was expressed in the kidney at an intermediate level when compared with other organs (Fig. S1*A*).

**Figure 1:**
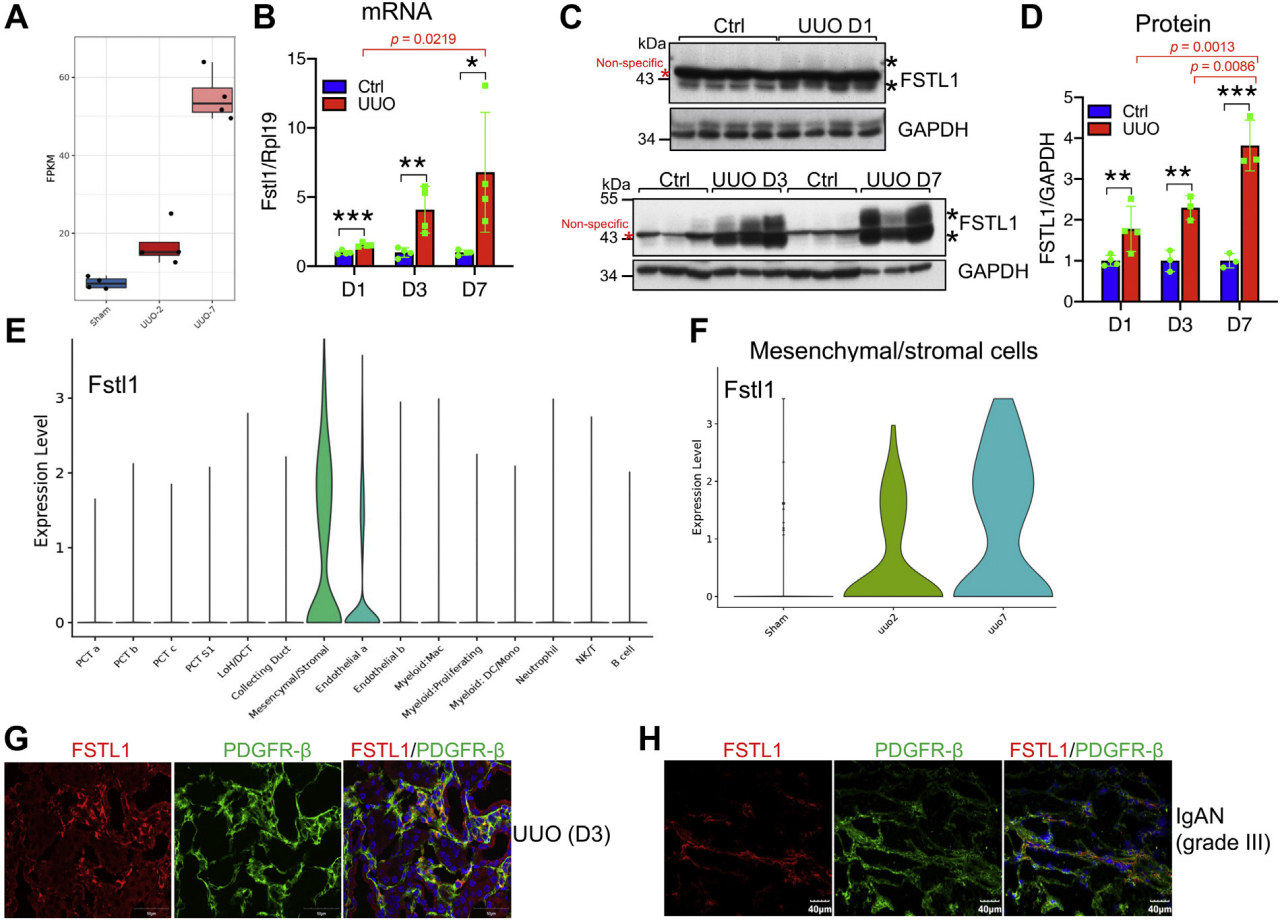
**Expression of FSTL1 in fibrogenic kidneys.***A*, Fstl1 expression in sham-operated and UUO kidneys. The data were extracted from the bulk RNA-Seq dataset (46). The *y*-axis shows fragments per kilobase per million mapped fragments (FPKM) (n = 4 per group). *B*–*D*, mRNA and protein levels of FSTL1 in the kidneys of mice subjected to UUO. Eight-week-old male mice were subjected to UUO on the left ureters. Mice were sacrificed 1, 3, and 7 days after UUO surgery. Right kidneys (Ctrl) and left kidneys (UUO) were collected and analyzed for Fstl1 mRNA levels (*B*) or for FSTL1 protein levels (*C*). Quantitative analysis of FSTL1 protein levels was performed by densitometry (*D*). *E* and *F*, violin plots of Fstl1 gene expression in different cell populations of UUO kidneys and in the mesenchymal/stromal cell cluster marked by Pdgfrb. The data were extracted from the single-cell RNA-Seq dataset on the renal cortex of UUO kidneys (46). The *y*-axis shows the log-scale normalized read count. *G* and *H*, cellular localization of FSTL1 in mouse UUO kidneys and human kidneys with IgA nephropathy (IgAN). Frozen sections of kidneys collected 3 days (D3) after UUO surgery (*G*) or IgAN kidneys at grade III (*H*) were subjected to immunofluorescent staining for FSTL1 (*red*) and PDGFR-β (*green*). *Red asterisks* indicate nonspecific bands, and *black asterisks* indicate FSTL1-specific bands. GAPDH was used as the loading control for Western blotting, and Rpl19 was used as the internal control for real-time PCR. n = 4 for *B*. ∗*p* < 0.05; ∗∗*p* < 0.01; and ∗∗∗*p* < 0.001. FSTL1, follistatin-like 1; IgA, immunoglobulin A; PDGFR-β, platelet-derived growth factor receptor beta; UUO, unilateral ureteral obstruction.

Unilateral ureteral obstruction (UUO) is a well-known mouse model of kidney fibrosis (45). We analyzed Fstl1 mRNA expression in UUO kidneys using the interactive tool for data exploration at http://www.ruuo-kidney-gene-atlas.com/ (46). Bulk RNA-Seq of the renal cortex showed that Fstl1 expression was induced by UUO (Fig. 1*A*). Interestingly, Fstl1 was also found to be increased in UUO kidneys in another RNA-Seq dataset (47). Consistently, our quantitative PCR analysis and Western blotting showed that Fstl1 mRNA (Fig. 1*B*) and protein (Fig. 1, *C* and *D*) levels in the whole kidney dramatically increased from day 1 to day 7 after UUO surgery.

We then extracted the data for Fstl1 from the single-cell RNA-Seq dataset from the renal cortex of UUO kidneys (46) and found that Fstl1 was most highly enriched in mesenchymal/stromal cells (Fig. 1*E*). Fstl1 enrichment in mesenchymal/stromal cells was found at UUO day 2 and increased at day 7 (Fig. 1*F*). In contrast, Fstl1 reads in endothelial cells were not enriched until UUO day 7 (Fig. S2).

As shown by immunofluorescence, FSTL1 was found in interstitial cells in kidneys with UUO for 3 days (Figs. 1*G* and S1*B*), whereas it was not detectable in control kidneys (Fig. S1*B*). FSTL1 was not detected in lymphocytes (Figs. S3*A* and 3*B*), macrophages (Fig. S3*C*), and dendritic cells (Fig. S3*D*). Interestingly, FSTL1 was colocalized with the fibroblast marker platelet-derived growth factor receptor beta (PDGFRβ) (Fig. 1*G*).

We also examined FSTL1 expression in kidneys from patients with immunoglobulin A nephropathy at grade III, or membranous nephropathy at grade II, which had developed renal fibrosis (data not shown), and found colocalization of FSTL1 with PDGFRβ (Figs. 1*H* and S4).

We then determined whether FSTL1 was induced by acute kidney injury (AKI). Both mRNA and protein levels of FSTL1 in the kidney were not altered by cisplatin treatment for 72 h (Figs. S5*A* and 5*B*). Fstl1 mRNA and protein levels were only slightly increased in kidneys 24 h after ischemia/reperfusion compared with the sham-operated control (Figs. S5*C* and 5*D*). These data indicate that FSTL1 expression was not or only slightly altered by cisplatin-induced or ischemia/reperfusion–induced AKI.

### FSTL1 overexpression aggravated renal fibrosis in obstructed kidneys

**Figure 2:**
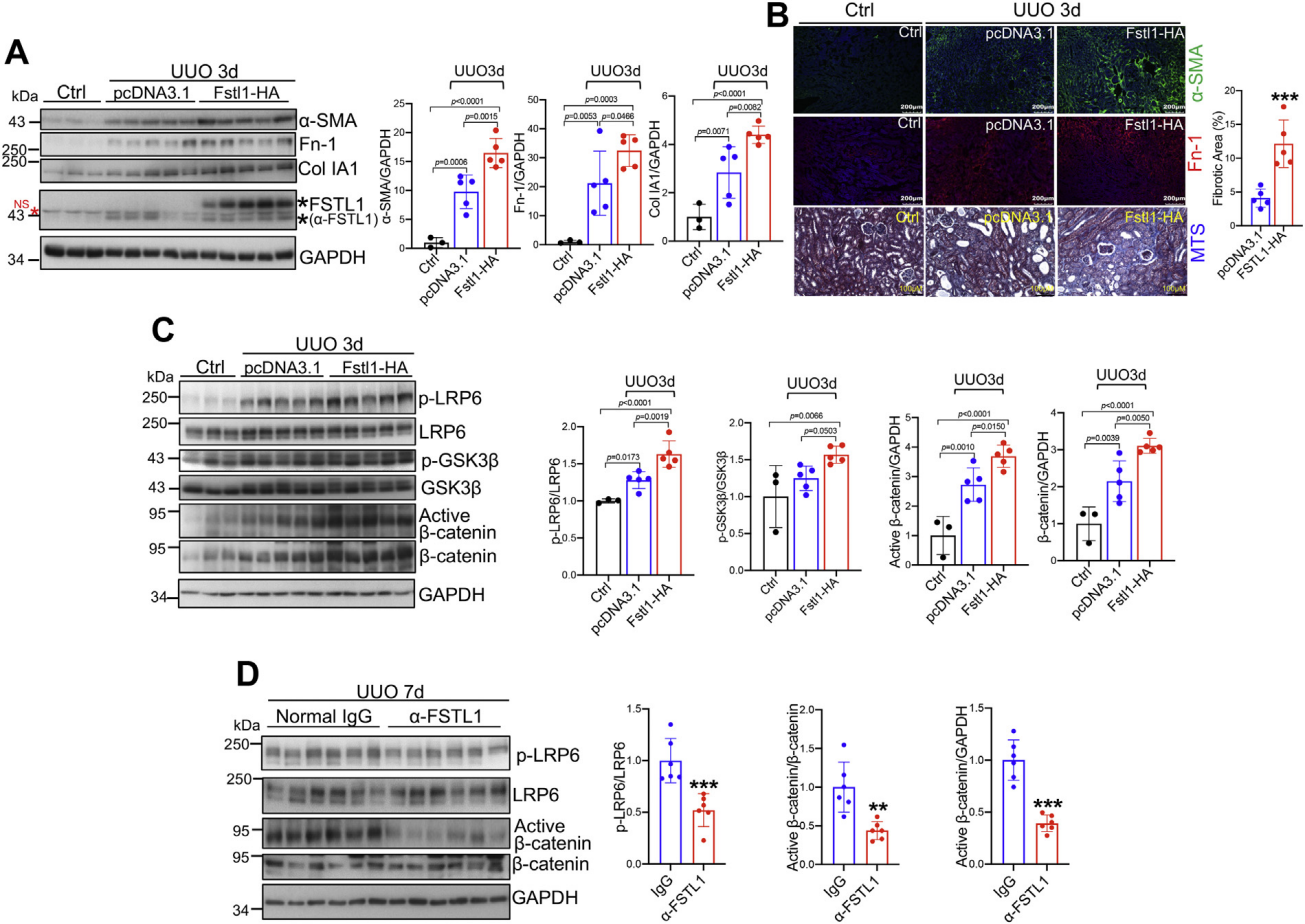
**Impacts of overexpression and neutralization of FSTL1 on renal fibrosis and on thecanonical Wnt/β-catenin pathways in UUO kidneys.***A*–*C*, effects of FSTL1 overexpression on renal fibrosis and on the Wnt/β-catenin pathway. Male mice at 8 weeks of age were subjected to UUO on left ureters. FSTL1-HA or pcDNA3.1 plasmid was injected *via* the tail vein on day 2 after UUO surgery. Mice were sacrificed the next day, and right (Ctrl) and left (UUO) kidneys were collected for analysis. *A*, kidney lysates were analyzed by Western blotting for FSTL1 expression and the fibrosis markers α-SMA, fibronectin-1 (Fn-1), and collagen 1 (Col IA1) (*left panel*). Levels of α-SMA, Fn-1, and Col IA1 relative to GAPDH are presented (*right panels*). *B*, immunofluorescence for α-SMA and Fn-1 and Masson’s trichrome staining (MTS) on sections from the UUO kidneys of mice injected with pcDNA3.1 or FSTL1-HA plasmids. Frozen sections were used for immunofluorescent staining for α-SMA (*green*) and Fn-1 (*red*). Paraffin sections were used for MTS. The *fibrotic blue area* was quantified. *C*, kidney lysates were analyzed by Western blotting for the Wnt/β-catenin pathway (p-LRP6, p-GSK3β, active β-catenin, and β-catenin) (*left panel*). Levels of p-LRP6 relative to LRP6; p-GSK3β relative to GSK3β; and active β-catenin and β-catenin relative to GAPDH are presented (*right panels*). *D*, effects of neutralization of FSTL1 on the Wnt/β-catenin pathway. Male mice at 8 weeks of age were subjected to UUO on left ureters. Normal goat IgG or goat anti-FSTL1 antibody was injected (i.p.) at 5 μg/g body weight 7 days after UUO surgery. About 6 h later, kidney samples were harvested: kidney lysates were analyzed by Western blotting for p-LRP6, active β-catenin, and β-catenin (*left panel*). Levels of p-LRP6 relative to LRP6 and active β-catenin relative to β-catenin and GAPDH are presented (*right panels*). ∗∗*p* < 0.01; ∗∗∗*p* < 0.001. FSTL1, follistatin-like 1; HA, hemagglutinin; IgG, immunoglobulin G; LRP6, low-density lipoprotein receptor–related protein 6; α-SMA, alpha-smooth muscle actin; TGF-β, transforming growth factor beta; UUO, unilateral ureteral obstruction.

To explore the role of FSTL1 in kidney fibrogenesis, we overexpressed FSTL1 in kidneys through hydrodynamic gene delivery. Fstl1-hemagglutinin (HA) or pcDNA3.1 plasmids were injected into mice *via* tail veins 2 days after UUO, and mice were sacrificed the next day. Western blotting confirmed FSTL1 overexpression in the kidneys of mice injected with Fstl1-HA plasmid (Fig. 2*A*). Alpha-smooth muscle actin (α-SMA), Fn-1, and type I collagen α1 levels in UUO kidneys were increased in Fstl1-HA–treated mice compared with control mice (Fig. 2*A*). Consistently, immunofluorescence also showed increases in the signals for Fn-1 and α-SMA by FSTL1 overexpression in UUO kidneys (Fig. 2*B*). Masson’s trichrome staining revealed that renal fibrosis was aggravated by FSTL1 (Fig. 2*B*). Interestingly, mRNA levels of the inflammatory factors Tnf-α (tumor necrosis factor alpha), Tgf-β1, Il-6 (interleukin 6), Il-1β (interleukin 1β), Mcp1, and Cxcl1 did not change by FSTL1 overexpression (Fig. S6). These data suggest that FSTL1 promoted fibroblast activation and fibrosis in UUO kidneys.

### FSTL1 overexpression promoted Wnt/β-catenin signaling in obstructed kidneys

FSTL1 was found to regulate TGF-β (27) and BMP (26) activities, both of which play key roles in kidney fibrosis (3). Surprisingly, TGF-β1 and Smad3 phosphorylation levels did not change by FSTL1 overexpression in UUO kidneys (Fig. S7). Smad1/5/8 phosphorylation levels were not altered either by FSTL1 (Fig. S7). The Notch (48), Hedgehog (49, 50), YAP, and Wnt (8) pathways also promote kidney fibrosis. However, the activities of Notch (Fig. S8*A*), Hedgehog (Fig. S8*B*), and YAP (Fig. S8*C*) were not altered by FSTL1 overexpression. Interestingly, phospho-LRP6 (S1490, a hallmark of Wnt pathway activation (51)), phospho-GSK3β, active (nonphospho) β-catenin, and total β-catenin were induced by UUO and were further elevated by FSTL1 overexpression (Fig. 2*C*). Therefore, the Wnt/β-catenin pathway was more activated by FSTL1 overexpression in UUO kidneys.

Phospho c-Jun N-terminal kinase (p-JNK) and phospho-Ca^2+^/calmodulin–dependent protein kinase II (CaMKII) levels in UUO kidneys were not altered by FSTL1 (Fig. S8*D*). These results indicate that FSTL1 may not regulate noncanonical Wnt signaling in obstructed kidneys.

### Neutralization of FSTL1 inhibited Wnt/β-catenin signaling in obstructed kidneys

To further verify the role of FSTL1 in the canonical Wnt pathway, we injected FSTL1 antibody into mice 7 days after UUO surgery and collected kidneys 6 h later. This antibody recognizes mFSTL1 but not the closely related mFSTL3, and it did not recognize mWNT3a, mTGF-β1, and mActivin A either (Fig. S9). Neutralization of FSTL1 lowered phospho-LRP6 and active β-catenin levels (Fig. 2*D*) but did not alter the TGF-β1 levels and the phosphorylation levels of Smad3 and Smad1/5/8 (Fig. S10), JNK and CaMKII (Fig. S11). These results support that FSTL1 promoted the Wnt/β-catenin pathway and had no effects on TGF-β, BMP, and noncanonical Wnt pathways in obstructed kidneys.

### FSTL1 promoted Wnt/β-catenin signaling and fibroblast activation *in vitro*

**Figure 3:**
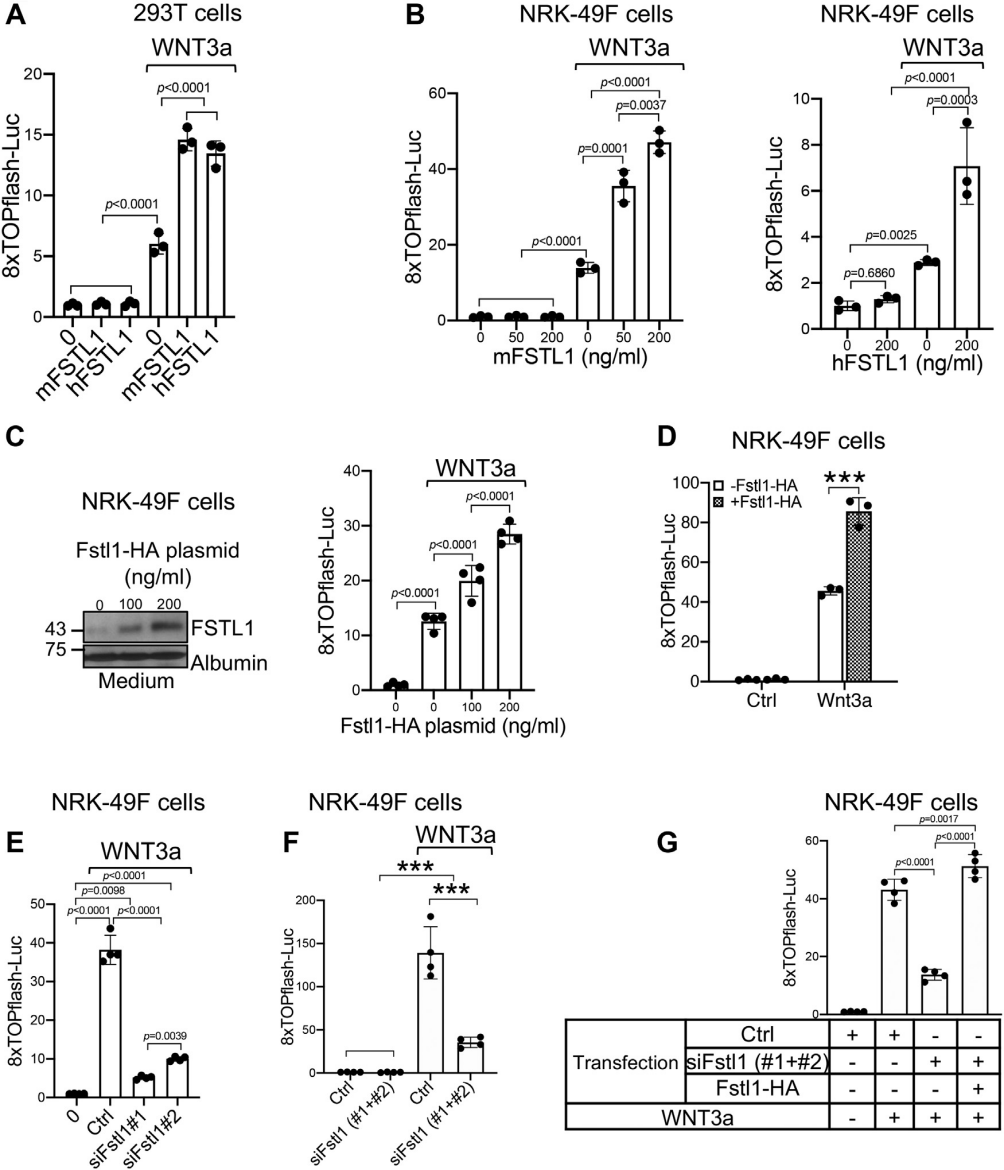
**FSTL1 enhances canonical Wnt signaling.***A*, both mFSTL1 and hFSTL1 proteins augmented mWnt3a mediated 8× TOPflash luciferase reporter activity in HEK293T cells. Cells were transfected with 8× TOPflash luciferase reporter and pRL-TK *Renilla* plasmids. Cells then were treated with and without mWnt3a protein (20 ng/ml) in the absence or the presence of mFSTL1 or hFSTL1 (200 ng/ml) before luciferase assay was performed. *B*, both mFSTL1 and hFSTL1 proteins promoted mWnt3a-mediated 8× TOPflash luciferase reporter activity in NRK-49F cells. Cells were transfected with 8× TOPflash luciferase reporter and pRL-TK *Renilla* plasmids. Cells then were treated with and without mWnt3a protein (20 ng/ml) in the presence of increasing amounts of mFSTL1 protein (*left panel*) or treated with and without mWnt3a protein (10 ng/ml) in the absence or the presence of hFSTL1 protein (*right panel*), before luciferase assay was performed. *C*, transfected FSTL1 promoted mWnt3a-induced 8× TOPflash luciferase reporter activity in a dose-dependent manner. NRK-49F cells were cotransfected with 8× TOPflash luciferase reporter and pRL-TK *Renilla* plasmids and increasing doses of FSTL1-HA plasmid. Cells then were treated with or without mWnt3a protein (15 ng/ml) before luciferase assay was performed (*right panel*). The *left panel* shows FSTL1 expression in cell culture medium. Albumin was used as loading control. *D*, transfected FSTL1 potentiated transfected Wnt3a-induced 8× TOPflash luciferase reporter activity. NRK-49F cells were cotransfected with 8× TOPflash luciferase reporter and pRL-TK *Renilla* plasmids, either alone or with FSTL1-HA plasmid, in the absence or the presence of Wnt3a plasmid. About 24 h after transfection, cells were serum starved for 16 h before luciferase assay was performed. *E* and *F*, siRNA-mediated inhibition of FSTL1 attenuated Wnt3a-induced 8× TOPflash luciferase reporter activity. NRK-49F cells were cotransfected with 8× TOPflash luciferase reporter and pRL-TK *Renilla* plasmids in the presence of scramble (Ctrl) or two Fstl1 siRNA sequences (siFstl1, 110 nM) either separately (*E*) or in combination (*F*). About 24 h after transfection, cells were incubated with or without Wnt3a protein (30 ng/ml) in serum-free medium for 16 h before cells were harvested for luciferase assay. *G*, the effects of siRNA-mediated inhibition of FSTL1 on Wnt3a signaling were rescued by FSTL1 overexpression. NRK-49F cells were cotransfected with 8× TOPflash luciferase reporter and pRL-TK *Renilla* plasmids in the presence of scramble (Ctrl) or Fstl1 siRNAs and with or without FSTL1-HA plasmid. About 24 h after transfection, cells were incubated with or without Wnt3a protein in serum-free medium for 16 h before cells were harvested for luciferase assay. n = 3 to 4. ∗∗∗*p* < 0.001. FSTL1, follistatin-like 1; HA, hemagglutinin; HEK293T, human embryonic kidney 293T cell line; hFSTL1, human FSTL1; mFSTL1, mouse FSTL1; NRK-49F, normal rat kidney 49 fibroblast cell.

To determine whether FSTL1 directly regulates Wnt signaling, we used the β-catenin-responsive TOPflash or 8× TOPflash luciferase reporter. Both mouse and human FSTL1 proteins increased mWNT3a protein-induced TOPflash-luciferase reporter activity in inner medullary collecting duct 3 (IMCD3) cells (Fig. S12*A*). As a control, mFSTL1 and hFSTL1 proteins inhibited BMP4-induced BRE luciferase reporter activity in IMCD3 cells (Fig. S12*B*). mFSTL1 and hFSTL1 proteins also promoted WNT3a-induced 8× TOPFlash-luciferase reporter activity in human embryonic kidney 293T (HEK293T) cells (Fig. 3*A*) and normal rat kidney 49F (NRK-49F) fibroblasts (Fig. 3*B*). These results indicate that FSTL1 enhanced WNT3a signaling in multiple kidney cell types.

To corroborate our findings from recombinant FSTL1 proteins, we overexpressed or knocked down FSTL1 in NRK-49F cells. Transfected Fstl1-HA dose-dependently enhanced 8× TOPflash luciferase reporter activity induced by WNT3a protein (Fig. 3*C*) or by transfected Wnt3a (Fig. 3*D*). Transfected Fstl1-HA also promoted Wnt/β-catenin signaling induced by transfected Wnt1, Wnt2, Wnt8a, Wnt9a, and Wnt10b (Fig. S13). Conversely, inhibition of FSTL1 expression by Fstl1 siRNA targeting (Fig. S14) decreased WNT3a protein-induced 8× TOPflash luciferase activity when two Fstl1 siRNA sequences were used either separately (Fig. 3*E*) or combinatorially (Fig. 3*F*), and this inhibition on Wnt3a signaling was fully rescued by cotransfection with Fstl1-HA (Fig. 3*G*).

**Figure 4:**
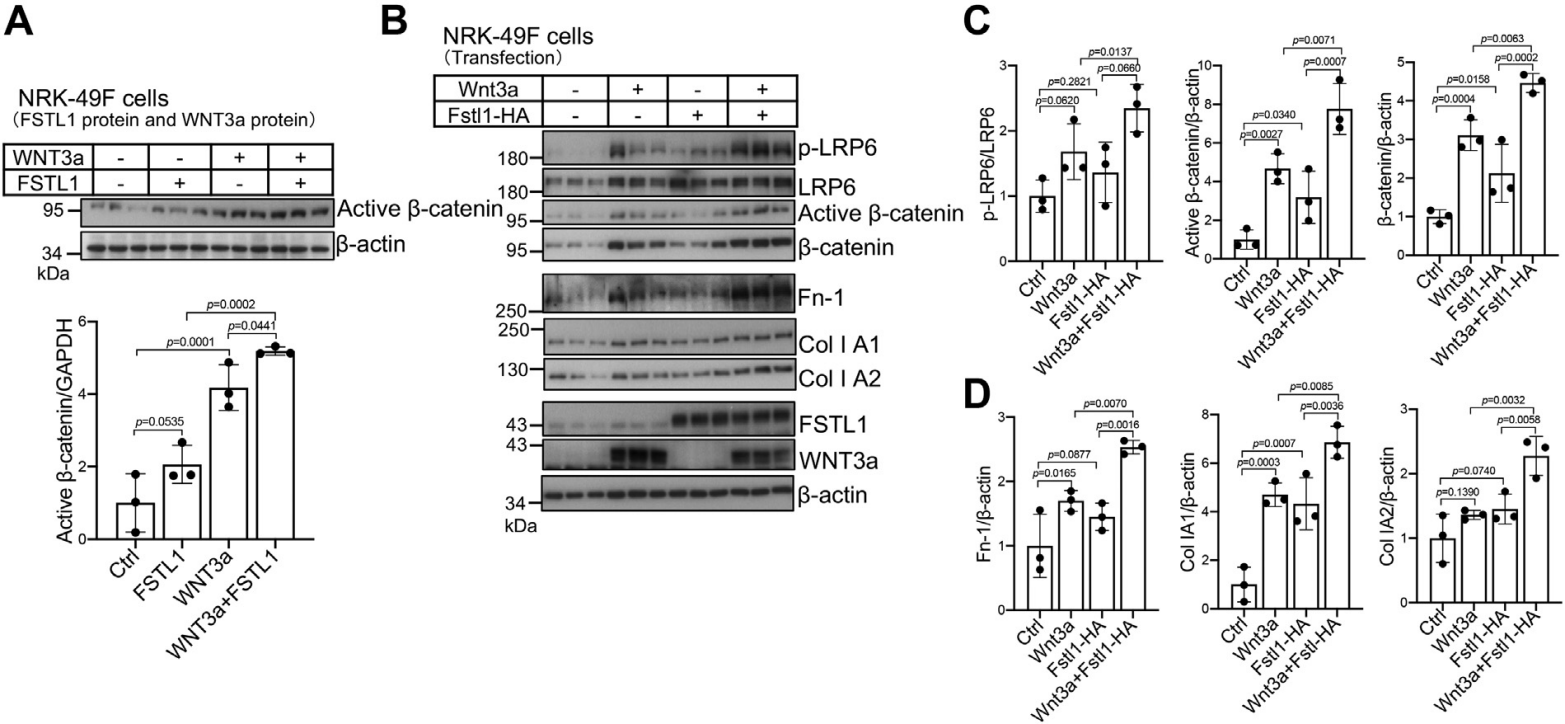
**FSTL1 activates β-catenin and fibroblasts.***A*, FSTL1 protein elevated active β-catenin levels induced by Wnt3a proteins in NRK-49F cells. FSTL1 protein (500 ng/ml) and Wnt3a (15 ng/ml) were preincubated overnight before they were added to serum-starved NRK-49F cells for 3 h. Cell lysates were used for Western blotting for active β-catenin levels (*upper panel*). Levels of active β-catenin relative to GAPDH are presented (*lower panel*). *B*–*D*, transfected FSTL1-HA promoted β-catenin levels and fibroblast activation induced by transfected Wnt3a in NRK-49F cells. Cells were transfected with Wnt3a plasmid (50 ng/ml) in the presence or the absence of FSTL1-HA plasmid (200 ng/ml). Cells were serum starved for 24 h before cells were harvested for Western blotting for p-LRP6, LRP6, active β-catenin, β-catenin, fibronectin (Fn-1), collagens (Col 1A1 and Col 1A2), FSTL1, and Wnt3a (*B*). Levels of p-LRP6 relative to LRP6, active β-catenin relative to β-actin, and β-catenin relative to β-actin were presented (*C*). Levels of Fn-1, Col 1A1, and Col 1A2 relative to β-actin were also presented (*D*). FSTL1, follistatin-like 1; HA, hemagglutinin; NRK-49F, normal rat kidney 49 fibroblast cell.

To determine whether FSTL1-induced 8× TOPflash activation correlates with β-catenin activation, we examined active β-catenin levels in NRK-49F cells. WNT3a protein increased active β-catenin levels, which were further elevated by cotreatment with FSTL1 protein (Fig. 4*A*). Transfected Fstl1-HA also increased the phospho-LRP6, active β-catenin, and total β-catenin levels induced by transfected Wnt3a (Fig. 4, *B* and *C*). These data indicate that FSLT1 promoted Wnt/β-catenin activation.

FSTL1 overexpression also promoted the expression of Fn and collagens induced by Wnt3a overexpression (Fig. 4, *B* and *D*). Therefore, FSTL1 stimulated fibroblast activity *in vitro*.

### FSTL1 interacted with Wnts

**Figure 5:**
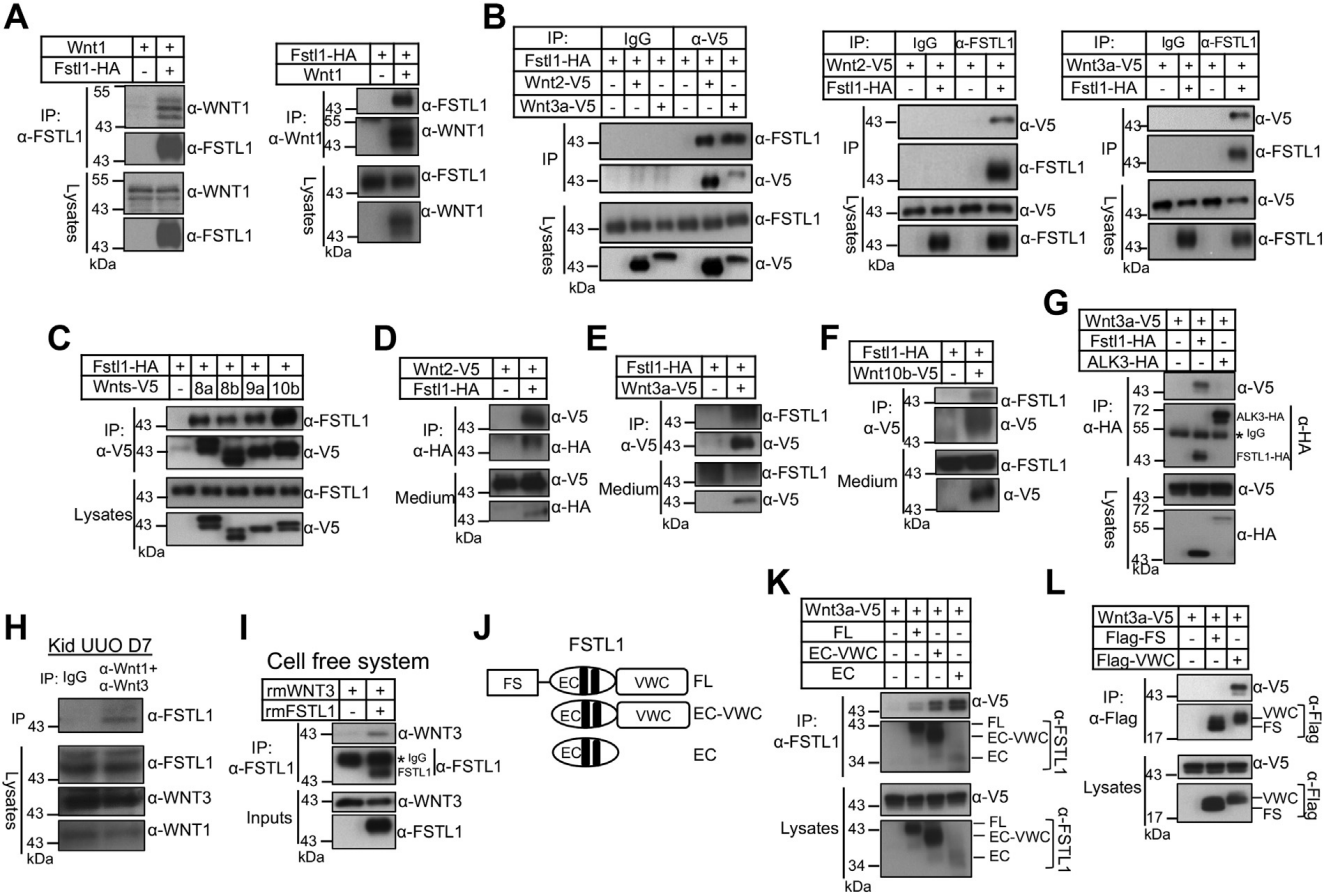
**FSTL1 interacts with canonical Wnt ligands.***A*, FSTL1 interacts with Wnt1. HEK293T cells were transfected with Wnt1 plasmid in the absence or the presence of FSTL1-HA plasmid. Cell lysates were immunoprecipitated with anti-FSTL1 to pull down Wnt1 (*left panel*). Reciprocal IP was performed (*right panel*). Western blotting was performed on whole lysates and precipitates as indicated. *B*, FSTL1 interacts with Wnt2 and Wnt3a. HEK293T cells were transfected with FATL1-HA in the absence or the presence of Wnt2-V5 or Wnt3a-V5 plasmid. Cell lysates were immunoprecipitated with IgG or anti-V5 antibody to pull down FSTL1 (*left panel*). Reciprocal IP was also performed (*right panels*). *C*, FSTL1 interacts with Wnt8a, Wnt8b, Wnt9a, and Wnt10b. HEK293T cells were transfected with FSTL1-HA in the absence or the presence of V5-tagged Wnt8a, Wnt8b, Wnt9a, and Wnt10b plasmids. Cell lysates were immunoprecipitated with anti-V5, followed by Western blotting with anti-FSTL1 or anti-V5 antibody on the precipitates and lysates as indicated. *D*, interaction of FSTL1 and Wnt2 occurs in the extracellular space. HEK293T cells were transfected with Wnt2-V5 in the absence or the presence of FSTL1-HA plasmid. Cell culture medium was immunoprecipitated with anti-HA antibody, followed by Western blotting with anti-V5 or anti-HA antibody on the precipitates and medium as indicated. *E* and *F*, interaction of FSTL1 and Wnt3a (*E*) or Wnt10b (*F*) occurs in the extracellular space. HEK293T cells were transfected with FSTL1-HA in the absence or the presence of Wnt3a-V5 (*E*) or Wnt10b-V5 (*F*) plasmid. Cell culture medium was immunoprecipitated with anti-V5 antibody, followed by Western blotting with anti-V5 or anti-FSTL1 on the precipitates and medium as indicated. *G*, ALK3 does not interact with Wnt3a. HEK293T cells were transfected with Wnt3a-V5 in the absence or the presence of FSTL1-HA or ALK3-HA plasmid. Cell lysates were immunoprecipitated with anti-HA antibody, followed by Western blotting with anti-V5 or anti-HA antibody on the precipitates and lysates as indicated. *H*, FSTL1 interacts with Wnt1 and Wnt3a in the kidney. Lysates from mouse kidneys with UUO for 7 days were immunoprecipitated with a combination of anti-Wnt1 and anti-Wnt3a antibodies or normal IgG, and the precipitates and lysates were subjected to Western blotting as indicated. *I*, FSTL1 directly interacts with Wnt3a. Recombinant Wnt3a protein (1.5 μg) was incubated with and without recombinant FSTL1 protein (3 μg) in 300 μl Tris-buffered saline/0.1% Triton X-100 overnight at 4 °C. Input samples were collected, and remaining mixtures were incubated with anti-FSTL1 antibodies followed by Protein G agarose. Eluted proteins and inputs were analyzed by Western blotting as indicated. *J*, domain organization of full-length (FL) and mutant FSTL1 proteins. *K*, interactions of EC–VWC and EC domains of FSTL1 with Wnt3a. HEK293T cells were transfected with Wnt3a-V5 plasmid in the absence or the presence of FL, EC–VWC, or EC plasmid. Cell lysates were immunoprecipitated with anti-FSTL1. Western blotting was performed on whole lysates and precipitates as indicated. *L*, VWC but not FS domain of FSTL1 interacts with Wnt3a. HEK293T cells were transfected with Wnt3a-V5 plasmid in the absence or the presence of 3× FLAG-FS or 3× FLAG-VWC plasmid. Cell lysates were immunoprecipitated with anti-FLAG. Western blotting was performed on whole lysates and precipitates as indicated. EC, the extracellular calcium–binding domain; FS, the follistatin-like domain; FSTL1, follistatin-like 1; HA, hemagglutinin; HEK293T, human embryonic kidney 293T cell line; IgG, immunoglobulin G; IP, immunoprecipitation; UUO, unilateral ureteral obstruction; VWC, a domain with homology to von Willebrand factor type C domain.

To identify the molecular mechanisms used by FSTL1 to enhance Wnt signaling, we first determined whether FSTL1 interacts with Wnts. We cotransfected Fstl1-HA with plasmids for several canonical Wnts in HEK293T cells. Co-IPs on whole lysates indicated that FSTL1 interacted with Wnt1 (Fig. 5*A*), Wnt2, Wnt3a (Fig. 5*B*), Wnt8a, Wnt8b, Wnt9a, and Wnt10b (Fig. 5*C*). Interactions between FSTL1 and Wnt2, Wnt3a, or Wnt10b were also found in the conditioned media from HEK293T cells cotransfected with Fstl1-HA and Wnt2-V5, Wnt3a-V5, or Wnt10b-V5 (Fig. 5, *D*–*F*). As a negative control, the BMP type I receptor ALK3 did not interact with Wnt3a (Fig. 5*G*).

We also examined whether endogenous FSTL1 and Wnts interacted with each other in the kidney. We used kidneys with UUO for 7 days since they expressed high levels of FSTL1 (Fig. 1*F*) and WNT1 and WNT3a (Fig. S15). We had difficulty pulling down FSTL1 using single anti-WNT1 antibody or single anti-WNT3a antibody possibly because FSTL1 was distributed to various Wnt ligands so that the proportion of FSTL1 for each individual ligand was small. Nevertheless, we were able to pull down FSTL1 using a combination of anti-WNT1 and WNT-3a antibodies (Fig. 5*H*).

To further explore the interactions between FSTL1 and Wnts, recombinant WNT3a protein alone or a combination of WNT3a and FSTL1 proteins were incubated in a solution, and the complexes containing FSTL1 were pulled down with anti-FSTL1. Protein blotting of the elutes showed that WNT3a was precipitated with FSTL1 (Fig. 5*I*). These data demonstrate a direct interaction between FSTL1 and WNT3a.

**Figure 6:**
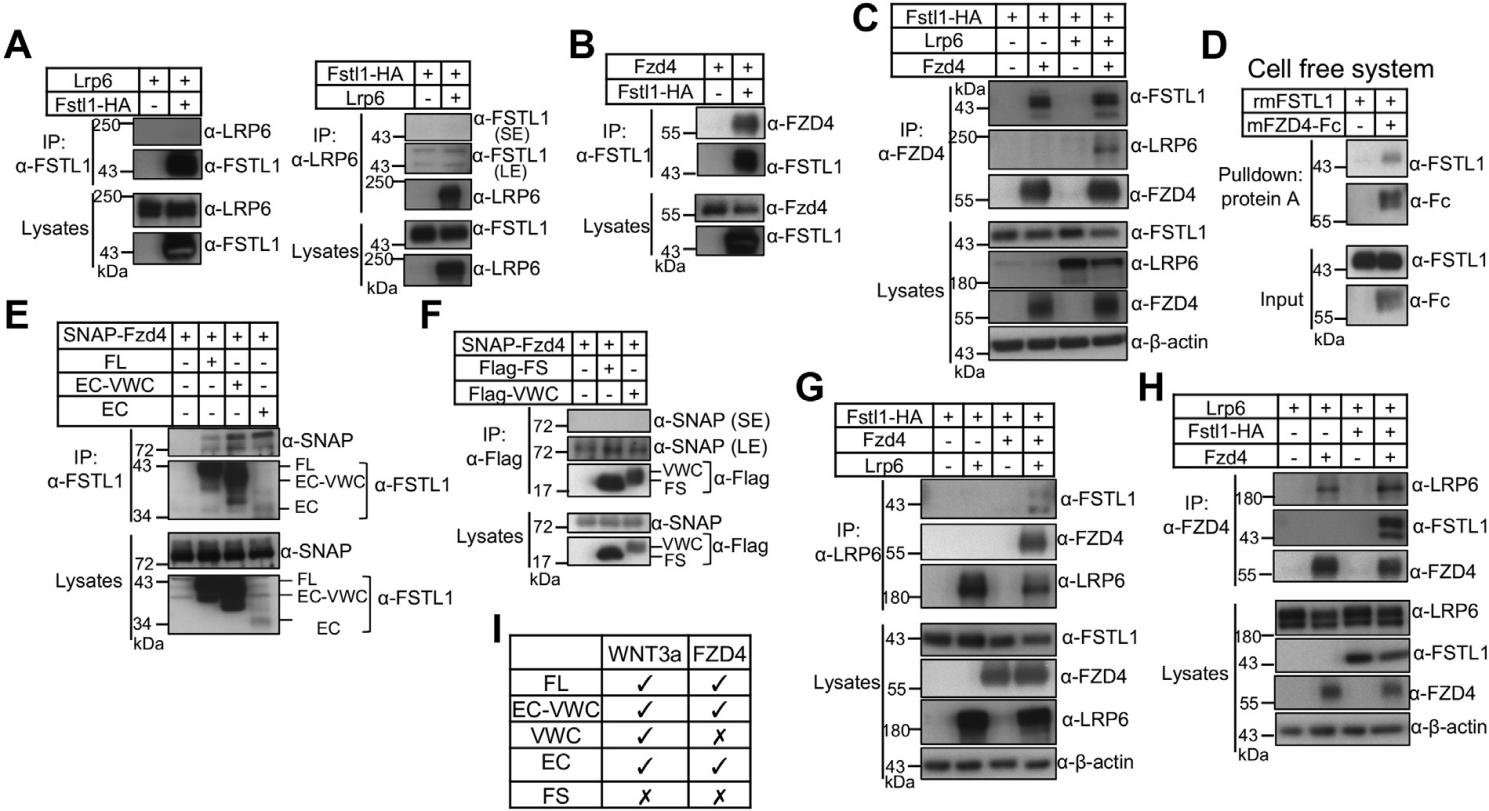
**FSTL1 interacts with FZD4 but not LRP6 and increases the association of Wnt3a with FZD4.***A*, FSTL1 does not interact with LRP6. HEK293T cells were transfected with LRP6 plasmid in the absence or the presence of FSTL1-HA plasmid. Cell lysates were immunoprecipitated with anti-FSTL1, and Western blotting was performed on whole lysates and precipitates as indicated (*left panel*). In reciprocal immunoprecipitation, HEK293T cells were transfected with FSTL1-HA plasmid in the absence or the presence of LRP-6 plasmid. Cell lysates were immunoprecipitated with anti-LRP6, and Western blotting was performed on whole lysates and precipitates as indicated (*right panel*). Short exposure (SE) did not show any FSTL1 band in the two lanes, and long exposure (LE) showed similar FSTL1 bands in density between the two lanes. *B*, FSTL1 interacts with FZD4. HEK293T cells were transfected with FZD4 in the absence or the presence of FSTL1-HA plasmid. Cell lysates were immunoprecipitated with anti-FSTL1 antibody to pull down FZD4. Western blotting was performed on whole lysates and precipitates as indicated. *C*, interaction of FZD4 and FSTL1 in the presence of LRP6. HEK293T cells were transfected with FSTL1-HA plasmid in the absence or the presence of FZD4 or LRP6 plasmids. Cell lysates were immunoprecipitated with anti-FZD4 antibody. Western blotting was performed on whole lysates and precipitates as indicated. *D*, FSTL1 directly interacts with FZD4. Recombinant FSTL1 protein (1 μg) was incubated with and without recombinant FZD4-Fc protein (3 μg) in 300 μl TBS/0.1% Triton X-100 overnight at 4 °C. Input samples were collected, and remaining mixtures were incubated with Protein A agarose. Eluted proteins and inputs were analyzed by Western blotting as indicated. *E*, interactions of EC–VWC and EC domains of FSTL1 with FZD4. HEK293T cells were transfected with SNAP-FZD4 plasmid in the absence or the presence of full-length (FL), EC–VWC, or EC plasmid. Cell lysates were immunoprecipitated with anti-FSTL1. Western blotting was performed on whole lysates and precipitates as indicated. *F*, neither VWC nor FS domain of FSTL1 interacts with Wnt3a. HEK293T cells were transfected with SNAP-FZD4 plasmid in the absence or the presence of FLAG-FS or FLAG-VWC plasmid. Cell lysates were immunoprecipitated with anti-FLAG. *G*, FSTL1, FZD4, and LRP6 form a complex. HEK293T cells were transfected with FSTL1-HA plasmid in the absence or the presence of FZD4 or LRP6 plasmid. Cell lysates were immunoprecipitated with anti-LRP6 antibody. Western blotting was performed on whole lysates and precipitates as indicated. *H*, interaction of FZD4 and LRP6 in the presence of FSTL1. HEK293T cells were transfected with LRP6 plasmid in the absence or the presence of FZD4 or FSTL1-HA plasmid. Cell lysates were immunoprecipitated with anti-FZD4 antibody. Western blotting was performed on whole lysates and precipitates as indicated. *I*, summary of the interactions between various FSTL1 domains with WNT3a and FZD4. FSTL1, follistatin-like 1; FZD, Frizzled; HA, hemagglutinin; HEK293T, human embryonic kidney 293T cell line; LE, long exposure; LRP, low-density lipoprotein receptor–related protein; SE, short exposure; SNAP-FZD4, SNAP-tagged FZD4; TBS, Tris-buffered saline.

FSTL1 consists of an FS domain and an EC domain, followed by a C-terminal domain with homology to the von Willebrand factor type C–like (VWC) domain (Fig. 5*J*) (52). IP assays demonstrated that the EC domain, VWC domain, or EC–VWC domains all pulled down WNT3a-V5, whereas the FS domain did not (Fig. 5, *K* and *L*), indicating that WNT3a interacted with the EC and VWC domains but not the FS domain (Fig. 6*I*). Together, our results suggest that FSTL1 interacted with various canonical Wnts. Given that Wnt proteins are highly conserved, our data also imply that FSLT1 interacts with different Wnt ligands through conserved structures of Wnt ligands.

### FSTL1 interacted with FZD4 but not LRP6 and enhanced WNT3a associations with FZD4

We then asked whether FSTL1 interacted with Wnt receptors. The Wnt receptor FZD4 and the coreceptor LRP6 are highly expressed in the kidney (Fig. S15) (53, 54). We therefore examined whether FSTL1 interacted with LRP6 and FZD4. As shown in Figure 6*A*, neither IP nor reciprocal IP detected any interaction between FSTL1 and LRP6. Interestingly, FSTL1-HA and FZD4 were able to pull down each other (Fig. 6, *B* and *C*). Both FSTL1-HA and FSTL1 that does not have a tag were coprecipitated with SNAP-tagged FZD4 (SNAP-FZD4) (Fig. S16). FSTL1 also was coprecipitated with FZD5 (Fig. S17). Pull-down assay using recombinant proteins showed a direct interaction between FZD4-Fc and FSTL1 (Fig. 6*D*). Using the constructs for various FSTL1 domains, we found that FZD4 interacted with the EC domain (Fig. 6*E*) but not with the FS and VWC domains (Fig. 6*F*), indicating FZD4 interacted with FSTL1 through the EC domain (Fig. 6*I*).

LRP6 did not apparently alter the interaction between FSTL1 and FZD4 (Fig. 6*C*, compare lanes 4 and 2 for FSTL1). In the presence of FZD4, LRP6 pulled down FZD4 and FSTL1 although LRP6 did not pull down FSTL1 in the absence of FZD4 (Fig. 6*G*), indicating that LRP6 pulled down FSTL1 through FZD4, and that FSTL1, FZD4, and LRP6 formed a complex. FSTL1 increased the interaction between FZD4 and LRP6 (Fig. 6*H*, compare lanes 4 and 2 for LRP6; Fig. S18, *A* and *B*, compare lanes 3 and 2 for LRP6).

**Figure 7:**
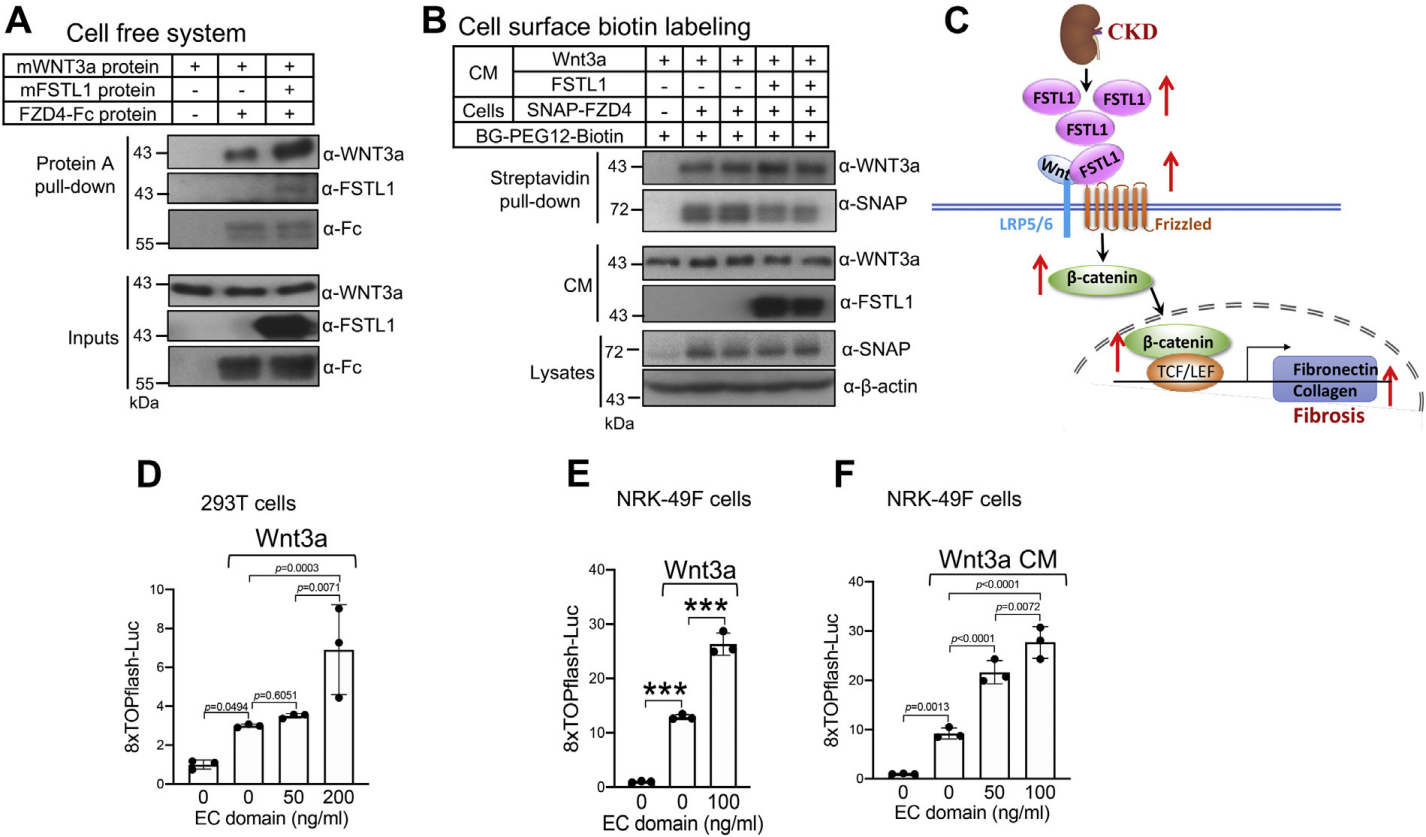
**FSTL1 increases the association of Wnt3a with FZD4.***A*, FSTL1 increases the level of Wnt3a pulled down by FZD4-Fc in a solution. Wnt3a protein (750 ng) was incubated overnight at 4 °C with and without FZD4-Fc protein (1 μg), and in the absence or the presence of FSTL1 protein (2 μg) in TBS buffer containing 0.1% Triton X-100 (500 μl). Input samples were collected, and remaining mixtures were incubated with protein A beads, which had been blocked with 0.1% BSA in TBS. Eluted proteins and inputs were analyzed by Western blotting as indicated. *B*, FSTL1 increases the binding of Wnt3a to FZD4 on cell surface. HEK293 cells were transfected with plasmids for SNAP-FZD4 (100 ng/ml) and the chaperone MESD (50 ng/ml). About 24 h after transfection, cells were serum starved overnight. Cells then were incubated for 3 h with Wnt3a conditioned medium (CM) in the absence or the presence of FSTL1-HA CM. Cells were incubated with 1 μM cell-impermeable biotinylated SNAP substrate (BG-PEG12-Biotin) for 15 min at room temperature. After washing with PBS, lysates were incubated with streptavidin–agarose, and the precipitates were used for Western blotting as indicated. *C*, working model showing how FSTL1 enhances renal fibrosis. In CKD, FSTL1 expression increases, and FSTL1 increases the presentation of Wnt ligands to FZD receptors, thus enhancing Wnt/β-catenin signaling and renal fibrosis. *D*–*F*, the EC domain enhances Wnt3a signaling. In *E* and *F*, HEK293T (*D*) or NRK-49F cells (*E*) were cotransfected with 8× TOPflash luciferase reporter and pRL-TK *Renilla* plasmids, either alone or with Wnt3a plasmid, in the absence or the presence of the plasmid for EC domain. In *F*, NRK-49F cells were cotransfected with 8× TOPflash luciferase reporter and pRL-TK *Renilla* plasmids, and increasing doses of EC plasmid before the cells were incubated with Wnt3a CM. ∗∗∗*p* < 0.001. BSA, bovine serum albumin; CKD, chronic kidney disease; EC, extracellular calcium–binding domain; FSTL1, follistatin-like 1; FZD, Frizzled; HA, hemagglutinin; HEK293 human embryonic kidney 293 cell line; MESD, mesoderm development LRP chaperone; NRK-49F, normal rat kidney 49 fibroblast cell; SNAP-FZD4, SNAP-tagged FZD4; TBS, Tris-buffered saline.

In a cell-free system, FZD4-Fc directly interacted with WNT3a, and FSTL1 enhanced this interaction (Fig. 7*A*). To investigate whether FSTL1 increases the association of WNT3a with FZD4 on the plasma membrane, we used SNAP-FZD4, which has been verified to be active in transducing Wnt/β-catenin signaling (55). SNAP-FZD4 allows for selective cell surface labeling of FZD4 by using BG-PEG12-Biotin, a membrane-impermeable biotinylated SNAP substrate (56). Streptavidin pulldown using lysates from HEK293T cell–expressing SNAP-FZD4 showed that FSTL1 enhanced the interactions between WNT3a and SNAP-FZD4 on the plasma membrane (Fig. 7*B*, compare lanes 4 and 5 with lanes 2 and 3 for WNT3a). Our results collectively show that FSTL1 interacted with Wnts and FZDs and promoted Wnt/β-catenin signaling by enhancing the presentation of Wnts to FZDs (Fig. 7*C*).

### The EC domain enhanced Wnt3a signaling

We have shown that the EC domain of FSTL1 interacted with both WNT3a and FZD4 (Fig. 6*I*). This property led us to examine whether the EC domain enhances Wnt signaling. As shown in Figure 7*D*, the EC domain dose-dependently increased transfected Wnt3a-induced luciferase activity in HEK293T cells. The EC domain also promoted Wnt signaling induced by Wnt3a transfection (Fig. 7*E*) or Wnt3a conditioned medium in NRK-49F cells (Fig. 7*F*). These results reinforce the notion that simultaneous binding of FSTL1 with Wnts and FZDs facilitates Wnt signaling.

### FSTL1 did not regulate TGF-β activities

We entertained our curiosity about whether FSTL1 enhances TGF-β1 signaling. Neither mFSTL1 nor hFSTL1 proteins augmented TGF-β1-induced CAGA luciferase activity in IMCD3 cells (Fig. S19, *A* and *B*). Overexpression (Fig. S19*C*) or inhibition (Fig. S19*D*) of FSTL1 did not alter TGF-β1-induced CAGA luciferase activity in NRK-49F cells either. FSTL1 also failed to alter TGF-β1-induced Smad3 phosphorylation and expression of Fn and collagen (Fig. S20). These results argue against a role of FSTL1 in TGF-β1 signaling.

## Discussion

In the present study, we provide evidence that FSTL1 promotes Wnt/β-catenin signaling and kidney fibrosis. FSTL1 has been found to promote fibrosis in the lung (27), heart (33), and liver (42). Consistently, in the present study, we identified a profibrotic role for FSTL1 in the kidney since overexpression of FSTL1 significantly increased the expression of the fibrotic proteins Fn and collagen I *in vitro* and *in vivo*. Previous studies identified both proinflammatory and anti-inflammatory roles for FSTL1 in human diseases and animal models (57). Interestingly, the inflammatory factors in the kidney remained unchanged after FSTL1 overexpression in UUO kidneys. Therefore, the increased fibrosis was not secondary to altered inflammation.

Activation of TGF-β1 and inadequate BMP signaling have been shown to play important roles in tissue fibrosis (58). FSTL1 was found to inhibit BMP signaling in lung epithelial cells during embryonic lung development (26). Dong *et al.* (27) demonstrated that FSTL1 facilitated TGF-β signaling in fibroblasts by interacting with TGF-β1 and TβRII. Based on these previous studies, we initially hypothesized that the profibrotic role of FSTL1 may be attributed to its role in TGF-β and BMP signaling. However, we failed to observe any changes in TGF-β and BMP signaling after overexpression or immunoneutralization of FSTL1 in UUO-induced fibrotic kidneys. In cell culture, we confirmed that FSTL1 inhibited BMP4 signaling but did not see any effects of FSTL1 overexpression or knockdown on TGF-β signaling and its fibrotic activity. Consistently, Nie *et al.* (30) failed to detect any interaction between FSTL1 and TGF-β1. FSTL1 had no effect on phospho-Smad2 and phospho-Smad3 in the presence or the absence of TGF-β1 stimulation in cardiac fibroblasts (28). FSTL1 did not alter phospho-Smad2/3 in fibroblast-like synoviocytes either (29). In line with these observations, FS and FSTL3, two other members of the FS-SPARC family, do not interact with TGF-β (23). All these results argue against a role of FSTL1 in regulating TGF-β signaling. Nevertheless, our results demonstrate that TGF-β signaling and BMP signaling did not mediate the profibrotic activity of FSTL1.

It has been well documented that Wnt/β-catenin signaling is activated during kidney injury and promotes renal fibrosis (5, 6, 8, 11). Interestingly, we found that FSTL1 interacted with various Wnt ligands, including Wnt1, Wnt2, Wnt3a, Wnt8a, Wnt8b, Wnt9a, and Wnt10b, which are concurrently upregulated in fibrotic kidneys after UUO. FSTL1 also interacted with FZD4 and FZD5 but not LRP6, and FSTL1 enhanced the interaction between FZD4 and Wnt3a. Furthermore, FSTL1 stimulated Wnt/β-catenin signaling induced by all Wnt ligands examined in several kidney cell types and promoted fibrogenesis induced by Wnt3a in NRK-49F fibroblasts. Overexpression of FSTL1 increased the expression of phospho-LRP6, active β-catenin, β-catenin, and phospho-GSK3 in UUO-induced fibrotic kidneys, whereas neutralization of FSTL1 inhibited phospho-LRP6 and active β-catenin levels. Since FSTL1 had no effect on other important fibrotic pathways, including the TGF-β, Notch, Hedgehog, and YAP pathways, it is likely that it is the Wnt pathway that mediates the profibrotic activity of FSTL1.

A previous study showed that FSTL1 promoted the Wnt/β-catenin–responsive TOPflash luciferase activity in breast cancer cells and that the FSTL1-induced TOPflash activity was inhibited by integrin β3 knockdown (59). This observation supports our present findings, but whether integrin β3 plays a role in the interactions between FSTL1 and Wnts or FZDs remains to be investigated.

We showed that FSTL1 interacted with Wnt3a through the EC and VWC domains and with FZD4 through the EC domain. The EC domain of FSTL1 is shared by Wnt3a and FZD4. Since FSTL1 enhanced the interaction of FZD4 and Wnt3a, it is conceivable that FZD4 and Wnt3a may contact FSTL1 at different regions of the EC domain. Future studies will be directed to identify the amino acids within the EC domain that are required for the interactions with Wnt3a and FZD4, respectively.

Our analysis on single-cell RNA-Seq of the renal cortex of UUO kidneys and immunofluorescence showed that FSTL1 was expressed in fibroblasts in fibrotic kidneys in mice and patients. These results are supported by a recent study, which analyzed publicly available small conditional RNA–Seq data from human kidneys with transplant nephropathy and found that FSTL1 was enriched in fibroblasts and myofibroblasts (60).

The baseline FSTL1 expression in the kidney is low, and cellular localization was not detectable by immunofluorescence. Therefore, the physiological role of FSTL1 in the kidney remains unknown. However, homozygous Fstl1 mutant carrying a gene trap showed no overt phenotype in the kidney despite dramatic reduction in FSTL1 expression (43). These results suggest that FSTL1 may not be essential for normal kidney function. In the same study, it was found that gene trap–induced FSTL1 downregulation exacerbated AKI induced by cisplatin, suggesting FSTL1 plays a protective role in AKI. It has been well documented that Wnt signaling plays dichotomous roles in kidney disease: it protects against tubular injury in AKI but promotes fibrosis in CKD (61). Therefore, it is possible that FSTL1 enhances Wnt signaling in renal tubules, whereby it protects against tubular injury during AKI.

Cardiac-specific Fstl1 deletion resulted in exacerbation of renal inflammation and tubulointerstitial fibrosis, whereas adenovirus-mediated systemic expression of FSTL1 ameliorated renal inflammation and tubulointerstitial fibrosis induced by subtotal (5/6) nephrectomy (44). These results suggest that FSTL1 exerts anti-inflammatory and antifibrotic effects in subtotal nephrectomy. What caused the discrepancy between this previous study and our present study is unknown. However, a recent study found that fibroblast-derived FSTL1 contributed to progression of CKD (60). Furthermore, FSTL1 was also found to promote fibrosis in lung (27), heart, (33) and liver (42). All these studies support a profibrotic role for FSTL1. In the future study, we will use fibroblast-specific Fstl1 knockout mice to confirm our findings.

In summary, we have demonstrated that FSTL1 is upregulated in fibroblasts in fibrotic kidneys. FSTL1 interacts with Wnts and enhances Wnt/β-catenin signaling by acting as a scaffold protein that holds Wnts and FZDs together. FSTL1 promotes the Wnt/β-catenin signaling pathway and thus aggravates renal fibrosis (Fig. 7*C*). Our study identifies FSTL1 as a novel positive regulator of Wnt/β-catenin signaling.

## Experimental procedures

### Mice

Bilateral ischemia–reperfusion injury and cisplatin-induced AKI and UUO were performed as previously described by us (62, 63, 64). Hydrodynamic gene delivery was performed 2 days after UUO surgery as described by Xiao *et al.* (13). About 20 μg Fstl1-HA plasmid or pcDNA3.1 (control plasmid) was diluted in 1.6 ml prewarmed saline and injected *via* the tail vein into the circulation within 5 to 10 s. Mice were sacrificed 3 days after UUO surgery.

FSTL1 antibody or nonspecific goat immunoglobulin G was injected intraperitoneally into mice at a dose of 5 μg/g body weight 7 days after UUO surgery. About 6 h after injection, kidney samples were collected.

### Histology and immunofluorescence

Paraffin kidney sections were used for Masson's trichrome staining to examine interstitial collagen deposits. Analysis of staining area was performed using ImageJ software (NIH).

Cryosections were used for immunofluorescent staining. Sections were treated with 1% SDS for 4 min. Sections then were incubated with anti-FSTL1 (catalog no.: AF1738 or AF1694; R&D Systems), anti-PDGFR-β (catalog no.: ab32570; Abcam), anti-CD45 (catalog no.: ab10558; Abcam), anti-CD3 (catalog no.: 11-0011-85; eBioscience), anti-CD4 (catalog no.: ab183685; Abcam), anti-F4/80 (catalog no.: 14-4801; eBioscience), anti-MHC2 (catalog no.: 70-5321; TONBO Biosciences), anti-α-SMA (catalog no.: ab5694; Abcam), and anti-Fn (catalog no.: AB2033; Millipore) antibodies individually or in combinations as indicated, followed by Alexa Fluor 546 or 555-labeled secondary antibodies (Invitrogen). Images were captured with a regular or confocal fluorescent microscope (Olympus FV1200).

### Construction of plasmids

Fstl1-HA, FLAG-Fzd4, and SNAP-Fzd4 plasmids were generously provided by Drs Cynthia M. Smas (65), Jorge Filmus (66), and Calvin J. Kuo (55), respectively, and myc-Lrp6, MESD (mesoderm development LRP chaperone), and SNAP-Fzd5 plasmids by Dr Madelon M. Maurice (56).

Plasmids for Fstl1, EC domain, and EC–VWC domain were already described (52). Plasmids for 3× FLAG-FS domain and 3× FLAG-VWC domain were generated using the p3× FLAG-CMV-9EXPRESION VECTOR (Sigma) and were sequenced and verified by Western blotting using anti-FSTL1 antibody and anti-FLAG antibody for their protein expression. Plasmids for Wnt ligands were either described previously (13) or purchased from Addgene.

### Cell culture and transfection

HEK293T cells and mouse IMCD3 cells were cultured in Dulbecco's modified Eagle's medium (DMEM) (high glucose) supplemented with 10% fetal bovine serum (FBS). NRK-49 fibroblasts were cultured in DMEM/F12 (1:1) medium supplemented with 10% FBS.

HEK293T cells were seeded in 6-well plates and transfected with Fstl1-HA in combination with plasmids for Wnt ligands using Lipofectamine 2000 (Invitrogen) according to the manufacturer’s instruction. Cells and media were collected for IP.

To study the effects of FSTL1 on the Wnt pathway, NRK-49F cells were seeded in a 12-well plate or 24-well plate and transfected with Fstl1-HA in the absence or the presence of plasmids for Wnt ligands. The transfected cells were starved with FBS-free DMEM/F12 medium containing 0.1% bovine serum albumin (BSA) for 20 h before the cells were harvested for analysis.

### IP

Cell lysates were incubated with antibodies as indicated in specific experiments at 4 °C overnight. The solutions were then incubated at 4 °C for 6 h with protein G or protein A beads (Pierce Biotechnology). Eluted proteins were subjected to Western blotting using antibodies as indicated.

To examine the interaction between endogenous FSTL1 and Wnt ligands in kidney, whole lysates from kidneys with UUO for 7 days were preabsorbed with protein G beads at 4 °C overnight. Preabsorbed lysates were incubated with rabbit anti-Wnt1 antibody (catalog no.: ab15251; Abcam) and rabbit anti-WNT3a antibody (catlog no.: 2721; Cell Signaling Technology) or normal rabbit immunoglobulin G at 4 °C overnight. Protein A beads were added, and lysates were incubated at 4 °C for 6 h. Eluted proteins were subjected to Western blot analysis using anti-FSTL1 antibody (catalog no.: AF1738; R&D Systems).

To determine whether there are direct interactions between FSTL1 and WNT3a, or between FZD4 and FSTL1, recombinant WNT3a (catalog no.: 315-20; PeproTech), FSTL1 (catalog no.:1738-FN; R&D Systems), and FZD4-Fc (catalog no.: 194-FZ; R&D Systems) proteins were added to Tris-buffered saline/0.1% Triton X-100 at indicated concentrations and incubated overnight at 4 °C. Input samples were collected, and remaining mixtures were incubated either with anti-FSTL1 antibodies (catalog no.: AF1738; R&D Systems) followed by Protein G agarose that had been blocked with 1% BSA for the FSTL1 and WNT3a interaction or with Protein A agarose that also had been blocked with 1% BSA for the FZD4-Fc and FSTL1 interaction. Eluted proteins and inputs were analyzed by Western blotting.

### Cell surface biotin labeling

For SNAP–biotin pulldowns, HEK293T cells were transfected with plasmids for SNAP-Fzd4 (100 ng/ml) and the chaperone MESD (50 ng/ml). About 24 h after transfection, cells were serum starved overnight before they were incubated for 3 h with Wnt3a conditioned medium in the absence or the presence of Fstl1-HA conditioned medium, which were also derived from HEK293T cells. Cells were incubated with 1 μM cell membrane–impermeable biotinylated SNAP substrate (BG-PEG12-Biotin; New England Biolabs) for 15 min at room temperature. After washing with PBS, lysates were incubated with streptavidin–agarose (Thermo Fisher Scientific) for 6 h, and the precipitates were analyzed by Western blotting for WNT3a and SNAP-FZD4.

### Western blotting

A total of 25 to 40 μg of protein was separated by SDS-PAGE and transferred to polyvinylidene difluoride membranes. Membranes were probed with anti-Wnt1 (catalog no.: ab15251; Abcam), anti-FSTL1 (catalog no.: AF1738; R&D Systems), anti-V5 (catalog no.: 13202; Cell Signaling Technology), anti-HA (catalog no.: 05-904; Millipore), anti-WNT3a (catalog no.: 2721; Cell Signaling Technology), anti-Myc (catalog no.: ab9106; Abcam), anti-SNAP (catalog no.: CAB4255; Thermo Fisher Scientific), anti-p-GSK3β (catalog no.: 9336; Cell Signaling Technology), anti-GSK3β (catalog no.: 9315; Cell Signaling Technology), anti-active (nonphospho) β-catenin (catalog no.: 8814; Cell Signaling Technology), anti-β-catenin (catalog no.: 8480; Cell Signaling Technology), anti-p-LRP6 (catalog no.: 2568; Cell Signaling Technology), anti-LRP6 (catalog no.: 3395; Cell Signaling Technology), anti-α-SMA (catalog no.: ab5694; Abcam), anti-Fn (catalog no.: AB2033; Millipore), anti-collagen 1 (catalog no.: bs-10423R; Bioss Antibodies), anti-TGF-β1 (catalog no.: bs-0086R; Bioss), anti-p-Smad3 (catalog no.: ab52903; Abcam), anti-Smad3 (catalog no.: 9523 or 9513; Cell Signaling Technology), anti-p-Smad1/5/8 (catalog no.: 9511; Cell Signaling Technology), anti-Smad1 (catalog no.: 9743; Cell Signaling Technology), anti-p-JNK (catalog no.: 9251; Cell Signaling Technology), anti-JNK (catalog no.: 9252; Cell Signaling Technology), anti-p-CaMKII (catalog no.: 12716; Cell Signaling Technology), and anti-CaMKII (catalog no.: ab52476; Abcam) antibodies. The signals were developed using Enhanced Chemiluminescence Western blotting detection reagents (Millipore or GE Healthcare).

### Luciferase assay and siRNA targeting

Mouse IMCD3, HEK293T, and NRK-49F cells were transiently transfected with a BMP-responsive reporter (BRE-Luc; BMP-responsive portion of the Id1 gene promoter), a TGF-β-responsive reporter ((CAGA)12MPL-Luc), or a β-catenin responsive TOPflash or 8× TOPflash luciferase reporter, in combination with pTK-RL (Promega), with or without cotransfection with FSTL1-HA plasmid, in the absence or the presence of plasmids for various Wnt ligands. Approximately 24 h after transfection, the medium was replaced with serum-free medium, with or without mFSTL1 (catalog no.: 1738-FN; R&D Systems) or hFSTL1 (catalog no.: 1694-FN; R&D Systems) protein, in the absence or the presence of mWNT3a protein (catalog no.: 1324-WN; R&D Systems). About 16 h later, cells were lysed, and luciferase activity was determined with the Dual Reporter Assay kits (Promega). Experiments were performed in triplicate or quadruplicate wells. Relative light units were calculated as ratios of firefly and Renilla luciferase values.

For Fstl1 targeting, NRK-49F cells were transfected (110 nM) with scrambled siRNA control (catalog no.: AM4613; Ambion) or a mixture of two rat fstl1 siRNA sequences (Shanghai GenePharma Co, Ltd): 5′-GCUGGAAGCCGAGAUCAUUTT-3’; 5′-GCCUCAACCCAUCCUUCAATT-3’.

### RNA isolation and real-time PCR

Total RNA was extracted using RNA extraction kit (catalog no.: 9767; Takara) with DNase I treatment. Reverse transcription was performed using PrimeScript RT reagent kit (catalog no.: RR036A; Takara). Real-time PCR was performed using SYBR Green PCR Master Mix (catalog no.: RR420D; Takara) on ABI Prism 7900 Sequence Detection System (PE Biosystems).

### Statistics

All data are represented as mean ± SD of three or more independent replicates. Comparisons between multiple groups were made by one-way ANOVA. Comparisons between two groups were made by Student’s *t* test. A *p* value of less than or equal to 0.05 was considered statistically significant. Representative results of one of three experiments were presented for luciferase assays, and representative results of one of two or three experiments were presented for IP.

### Study approval

All animal studies were approved by The Chinese University of Hong Kong Animal Experimentation Ethics Committee and were conducted in accordance with The Chinese University of Hong Kong animal care regulations. The use of human kidney samples was approved by Sun Yat-sen University Experimentation Ethics Committee.

## Data availability

All data are contained within the article. All original data pertaining to this study will be made available upon request.

## Supporting information

This article contains supporting information.

## Conflict of interest

The authors declare that they have no conflicts of interest with the contents of this article.
