# Supplementary material for: Follistatin-like 1 (FSTL1) interacts with Wnt ligands and Frizzled receptors to enhance Wnt/β-catenin signaling in obstructed kidneys in vivo
Source: J Biol Chem. 2022 May 4;298(7):102010. doi: 10.1016/j.jbc.2022.102010 (PMC9234244; doi:10.1016/j.jbc.2022.102010)
Supplement: Supplemental Figures S1–S20 [file mmc1.pdf]

Fig. S1

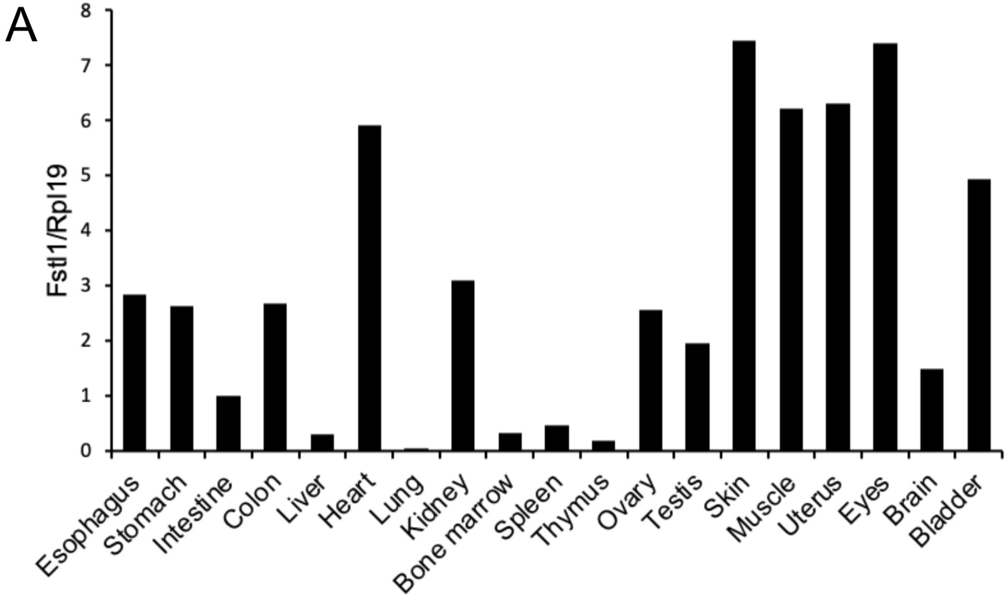

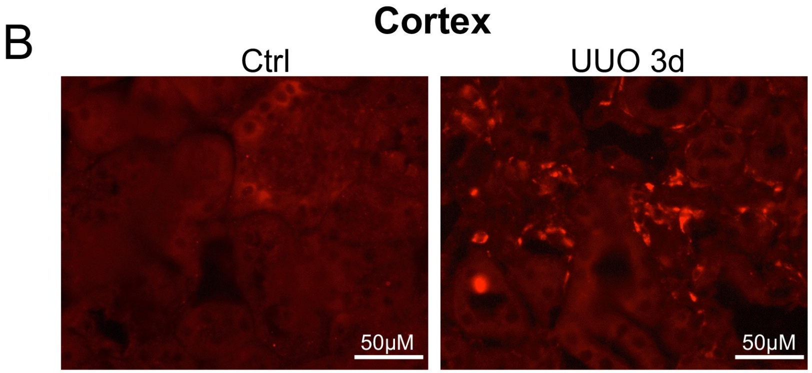

Fig. S1. Fstl1 mRNA expression in normal adult mice. (A) Different organs collected from 8-week-old male mice were analyzed for Fstl1 mRNA expression by real-time PCR. Rpl19 was used as the internal control. Samples from 3 mice were pooled. (B) Immunofluorescence for FSTL1 in control (Ctrl) kidneys and kidneys with UUO for 3 days (UUO 3d).

Fig. S2

Endothelial cells a

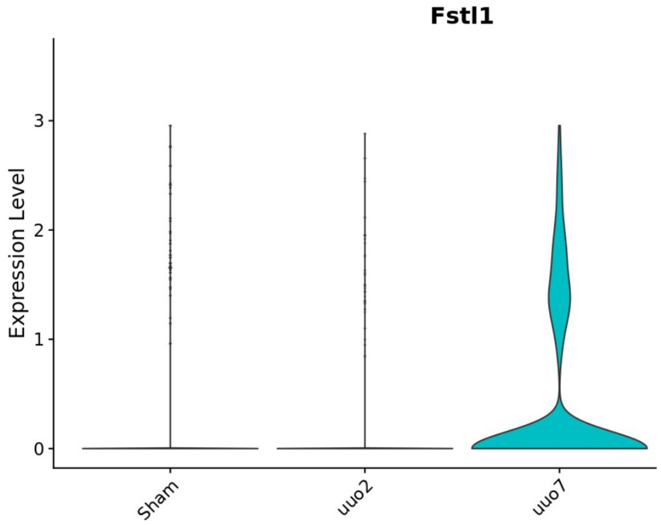

Fig. S2. Violin plots of Fstl1 gene expression in different cell populations in the kidney. The data were extracted from the single cell RNA-seq dataset on the renal cortex of UUO kidneys (Conway et al. *JASN* 2020). The y axis shows the log-scale normalized read count.

Fig. S3

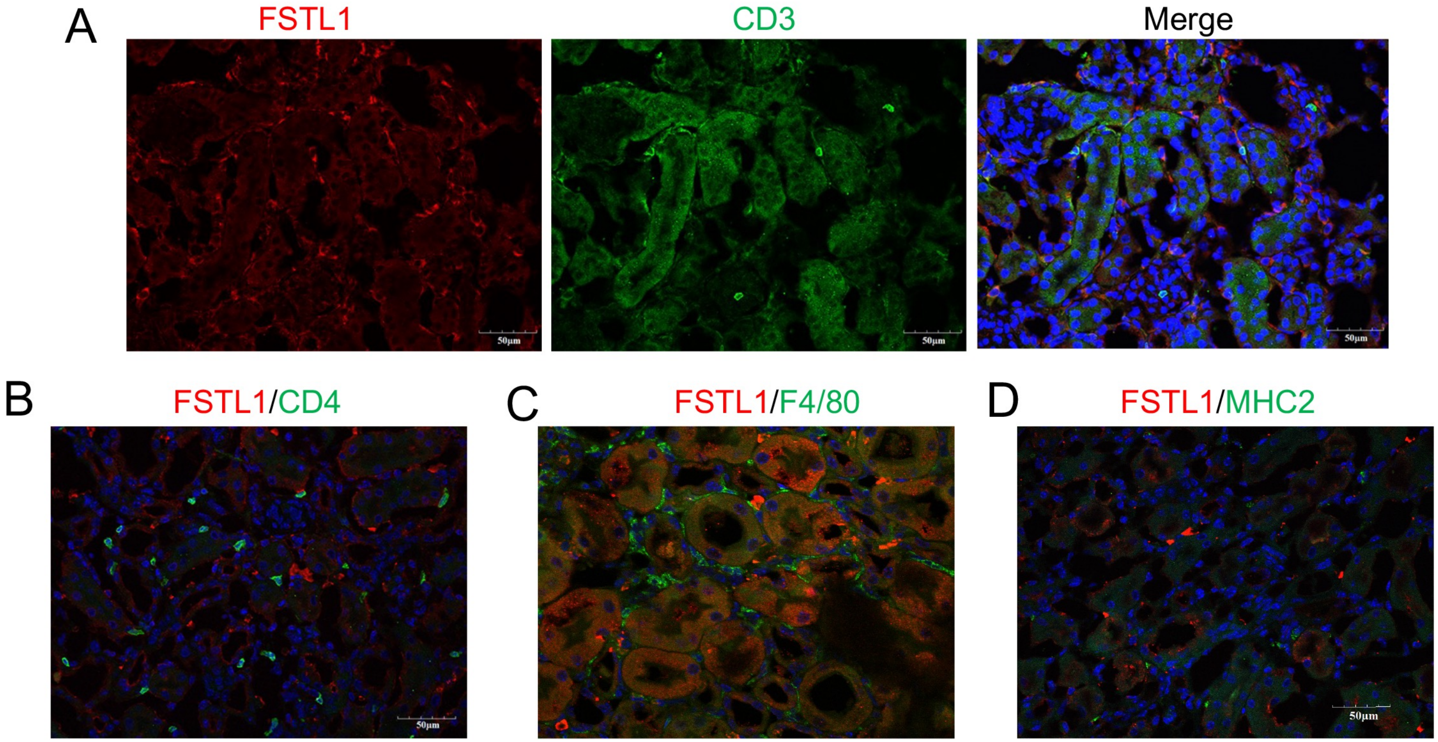

Fig. S3. Co-staining of FSTL1 and CD3, CD4, F4/80 or MHC2. Frozen sections of kidneys collected 3 days after UUO surgery were subjected to immunofluorescent staining for FSTL1 (red) and CD3 (green) (A), or CD4 (green) (B), or F4/80 (green) (C), or MHC2 (green) (D).

Fig. S4

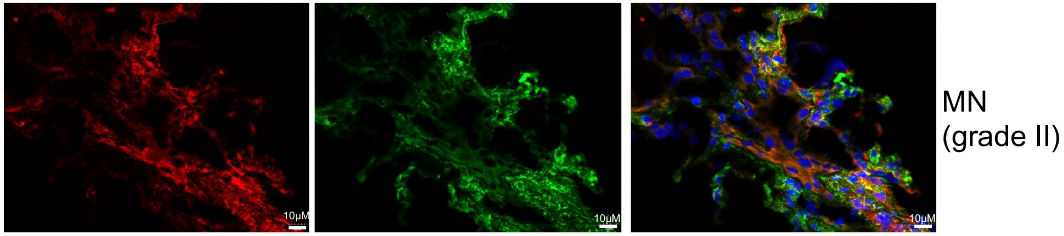

Fig. S4. Cellular localization of FSTL1 in human kidneys with membranous nephropathy (MN). Frozen sections of kidneys from human kidneys with MN at grade II were subjected to immunofluorescent staining for FSTL1 (red) and PDGFR-β (green).

Fig. S5

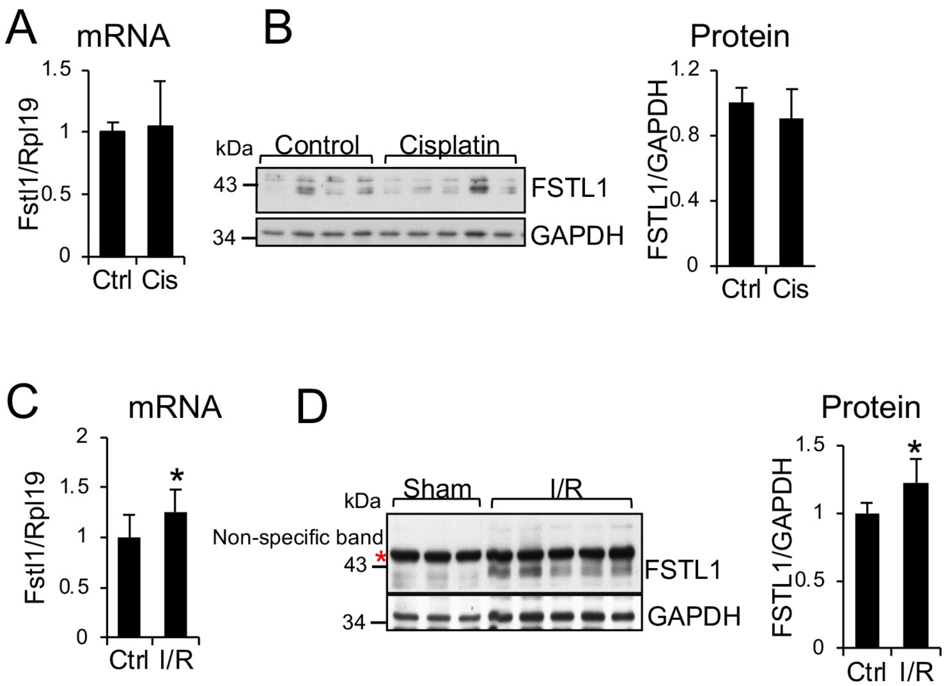

Fig. S5. Expression of FSTL1 in acute kidney injury. (A and B) mRNA and protein levels of FSTL1 in the kidneys of mice 72h after cisplatin injection (20 mg/kg body weight, i.p.). Kidneys collected from 8-week-old male mice injected with cisplatin or saline (control) were analyzed for Fstl1 mRNA levels by real-time PCR (A) or for FSTL1 protein levels by Western blotting (B, left panel). Quantitative analysis of FSTL1 protein levels was performed by densitometry (B, right panel). (C and D) mRNA and protein levels of FSTL1 in the kidneys of mice 24 h after bilateral I/R (40 min clamping). Kidneys collected from sham (control) and I/R mice were analyzed for Fstl1 mRNA levels (C) or for FSTL1 protein levels (D, left panel). Quantitative analysis of FSTL1 protein levels was performed by densitometry (D, right panel). n=4/9 (control/cisplatin) for panel A; n= 8 for panel C. *P<0.05.

Fig. S6

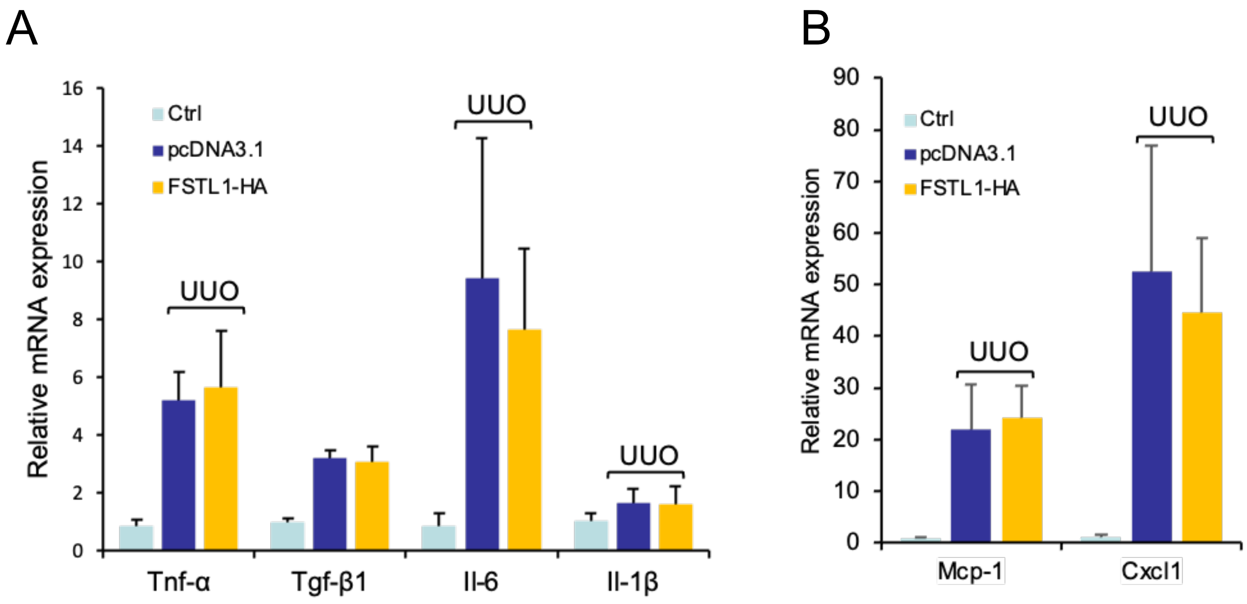

Fig. S6. Overexpression of FSTL1 did not change the inflammatory responses after UUO in mice. mRNA levels of Tnf-α, Tgf-β, Il-6, Il-1β (A), Mcp1, and Cxcl1 (B) are presented. Kidneys collected from control (contralateral right kidneys of pcDNA3.1 treated mice), pcDNA3.1-treated and FSTL-HA-treated UUO 3D mice were used for mRNA analysis by real-time PCR. n=5. Rpl19 was used as the internal control.

Fig. S7

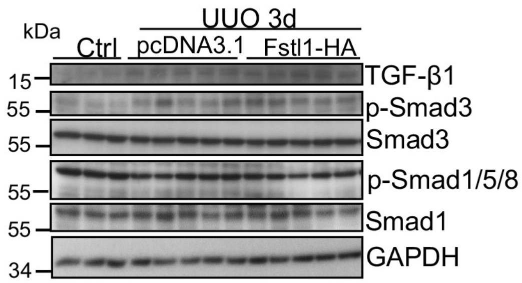

Fig. S7. Impacts of FSTL1 overexpression on the TGF-β and BMP pathways in UUO kidneys. Male mice at 8 weeks of age were subjected to UUO on left ureters. FSTL1-HA plasmid or pcDNA3.1 (control plasmid) was injected via the tail vein into the circulation on day 2 after UUO surgery. Mice were sacrificed 3 days after UUO surgery, and right (control) and left (UUO) kidneys were collected for analysis. Kidney lysates were analysed by Western blotting for TGF-β1, phospho-Smad3, Smad3, phospho-Smad1/5/8 and Smad1.

Fig. S8

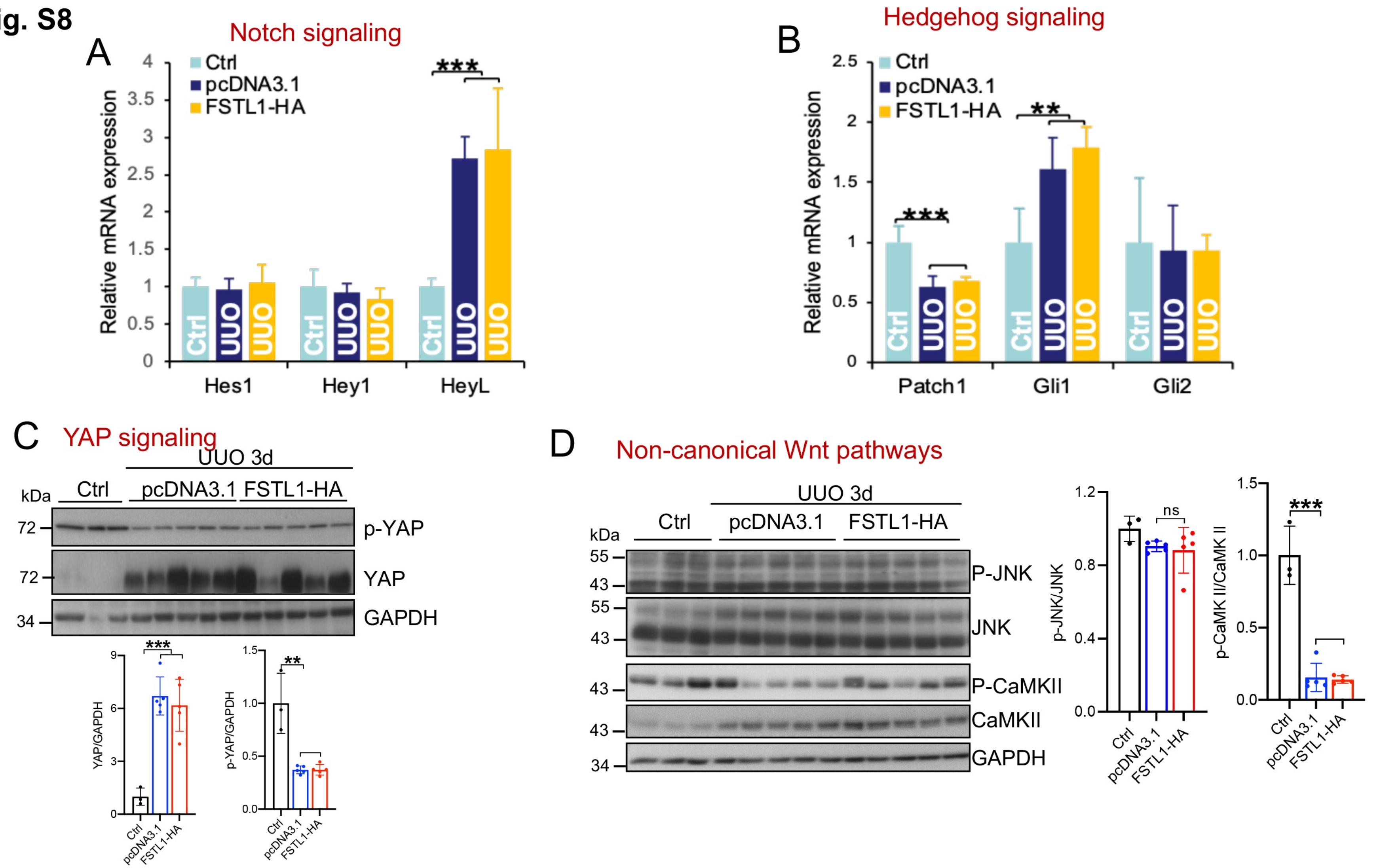

Fig. S8. Impacts of FSTL1 overexpression on the Notch, Hedgehog, YAP and non-canonical Wnt pathways in UUO kidneys. Male mice at 8 weeks of age were subjected to UUO on left ureters. FSTL1-HA plasmid or pcDNA3.1 (control plasmid) was injected via the tail vein into the circulation on day 2 after UUO surgery. Mice were sacrificed 3 days after UUO surgery, and right (control) and left (UUO) kidneys were collected for analysis. (A) Kidney lysates were analysed by real-time PCR for the mRNA levels of Hes1, Hey1 and HeyL, the markers for Notch signaling. (B) mRNA levels of Ptch1, Gli1 and Gli2, the markers for hedgehog signaling. (C) Kidney lysates were analysed by Western blotting for phospho-YAP and YAP levels (left panel). Levels of p-YAP and YAP relative to GAPDH are presented (right panel). (D) Kidney lysates were analysed by Western blotting for the non-canonical Wnt (P-JNK and P-CaMKII) pathways. n=5. ***P*<0.01; ****P*<0.001.

Fig. S9

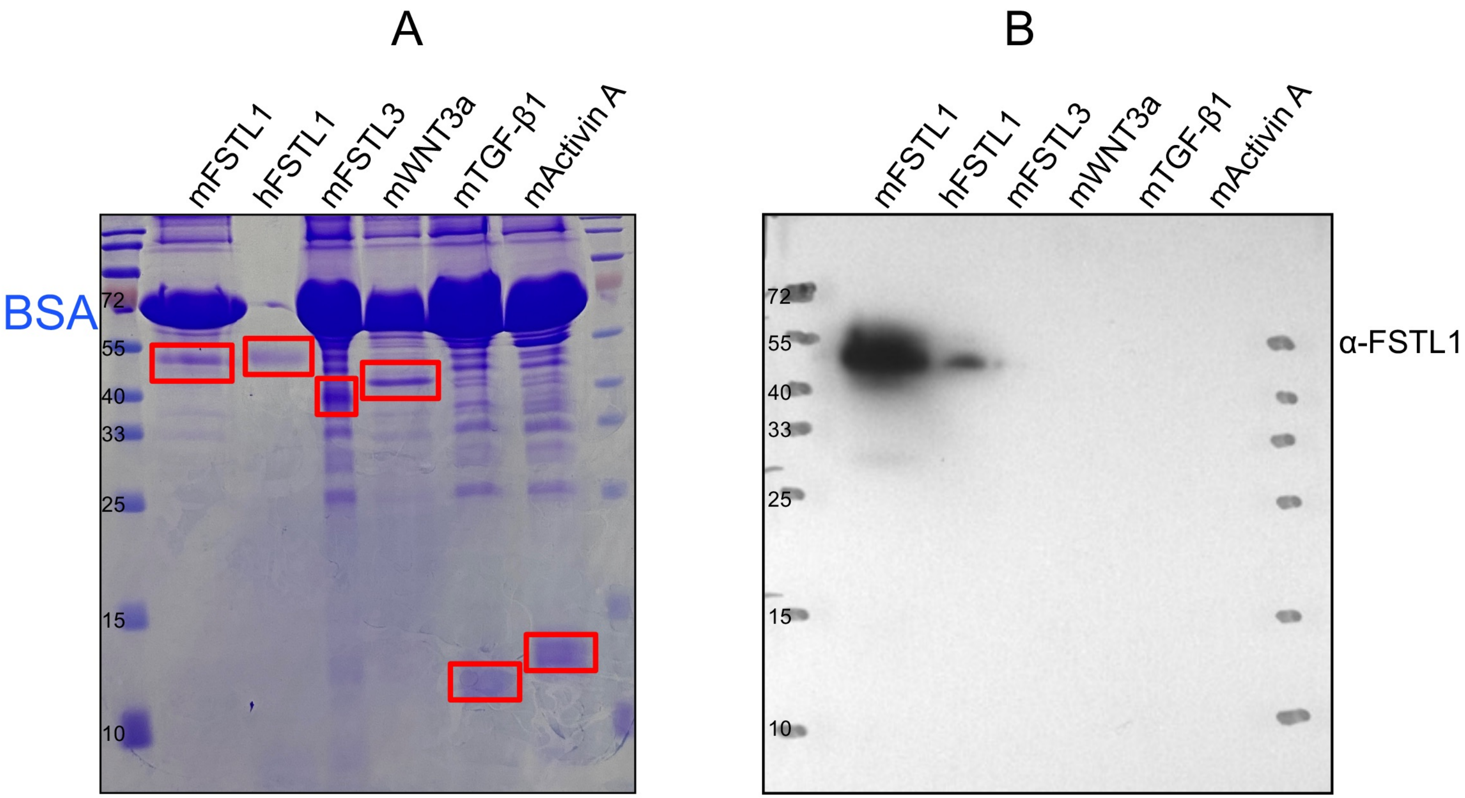

Fig. S9. Selectivity of the FSTL1 antibody. Recombinant mouse FSTL1, human FSTL1, mouse FSTL3, mouse WNT3a, mouse TGF- β1 and mouse Activin A proteins each at 200 ng were used for SDS-PAGE, followed by Coomassie Brilliant Blue staining (A) or by Western blotting using the FSTL-1 antibody (B). Red boxes in panel A indicate the locations of the respective proteins.

Fig. S10

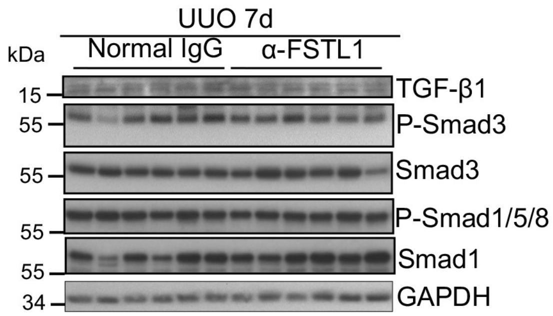

Fig. S10. Impacts of FSTL1 immunoneutralization on the TGF-β and BMP pathways in UUO kidneys. Male mice at 8 weeks of age were subjected to UUO on left ureters. Normal goat IgG or goat anti-FSTL1 antibody was injected (i.p.) at 5 μg/g body weight 7 days after UUO surgery. 6 h later, Kidney samples were collected. Kidney lysates were analysed by Western blotting for TGF-β1, phospho-Smad3, Smad3, phospho-Smad1/5/8 and Smad1.

Fig. S11

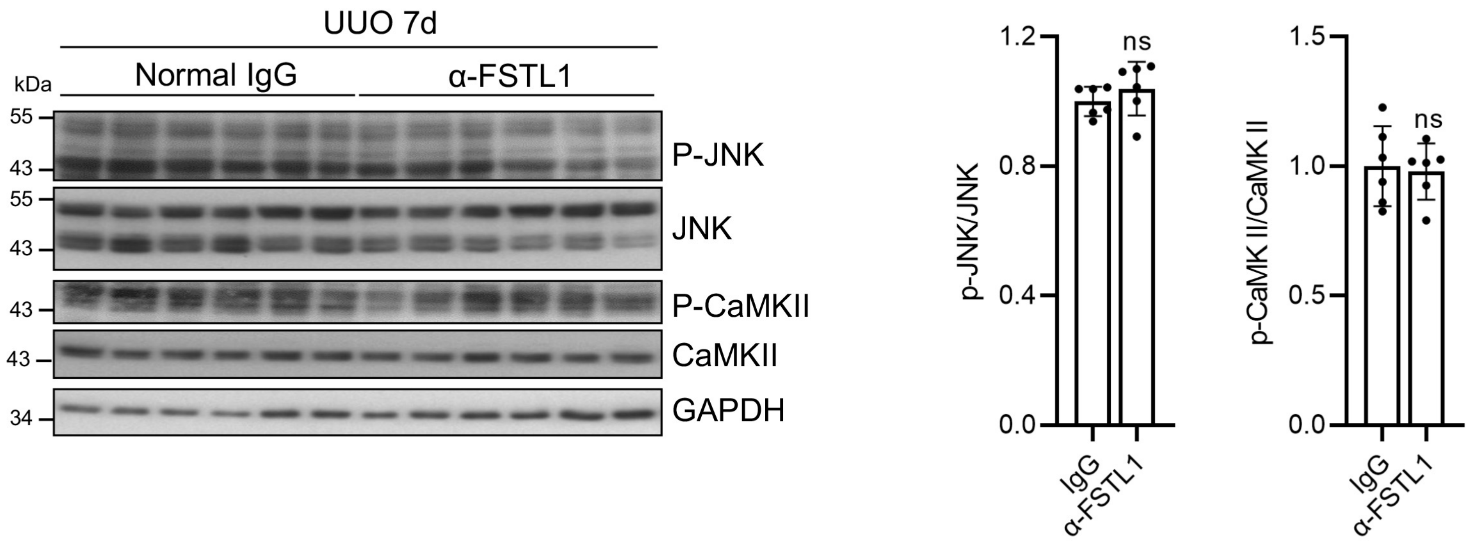

Fig. S11. Effects of neutralization of FSTL1 on the non-canonical Wnt/β-catenin pathway. Male mice at 8 weeks of age were subjected to UUO on left ureters. Normal goat IgG or goat anti-FSTL1 antibody was injected (i.p.) at 5 μg/g body weight 7 days after UUO surgery. 6 h later, Kidney samples were collected. Kidney lysates were analysed by Western blotting for the non-canonical Wnt (P-JNK and P-CaMKII) pathways.

Fig. S12

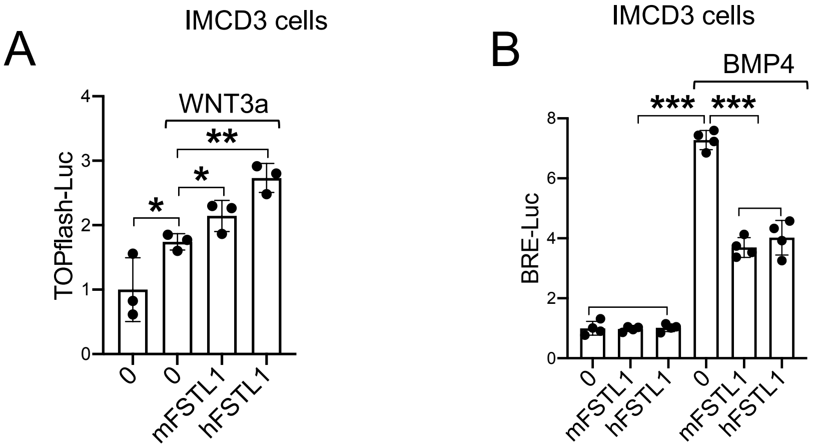

Fig. S12. FSTL1 enhances Wnt signaling in IMCD3 cells. (**A**) Both recombinant mFSTL1 and hFSTL1 proteins promoted mWnt3a mediated TOPflash luciferase reporter activity in IMCD3 cells. Cells were transfected with TOPflash luciferase reporter and pRL-TK Renilla plasmids. 24 h after transfection, cells were treated with and without mWnt3a (100 ng/ml) in the absence or presence of mFSTL1 or hFSLT1 (200 ng/ml) in serum-free media. 16 h later, cells were harvested for luciferase assay. (**B**) Both mFSTL1 and hFSTL1 decreased BMP-4 mediated BRE luciferase reporter activity in IMCD3 cells. Cells were transfected with BRE luciferase reporter and pRL-TK Renilla plasmids. Cells then were treated with and without BMP4 (20 ng/ml) in the absence or presence of mFSTL1 or hFSTL1 (200 ng/ml) before luciferase assay was performed. n=3-4. **P*<0.05; ***P*<0.01; ****P*<0.001.

Fig. S13

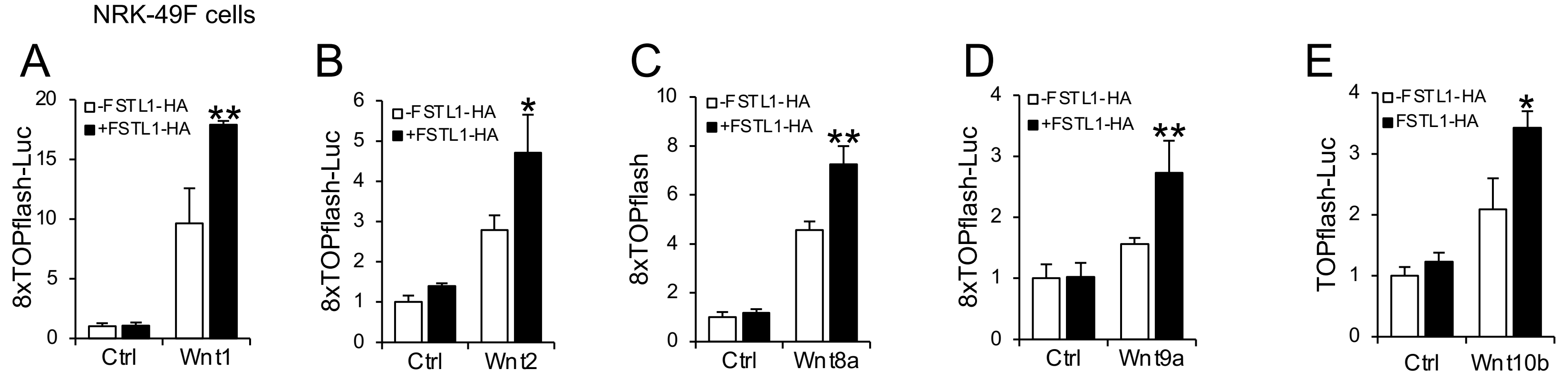

Fig. S13. Transfected FSTL1 promoted transfected Wnt1-, Wnt2-, Wnt8a-, Wnt9a- or Wnt10b-mediated 8×TOPflash luciferase reporter activity. NRK-49F cells were co-transfected with 8×TOPflash luciferase reporter plasmid and FSTL1-HA plasmid in the absence or presence of Wnt1-HA (A), Wnt2-V5 (B), Wnt8a-V5 (C), Wnt9a-V5 (D), or Wnt10b-V5 (E) plasmid. pTK-RL was included to control for transfection efficiency, and relative light units were calculated as ratios of firefly and Renilla luciferase values. n=3-4. *$P<0.05$; **$P<0.01$.

Fig. S14

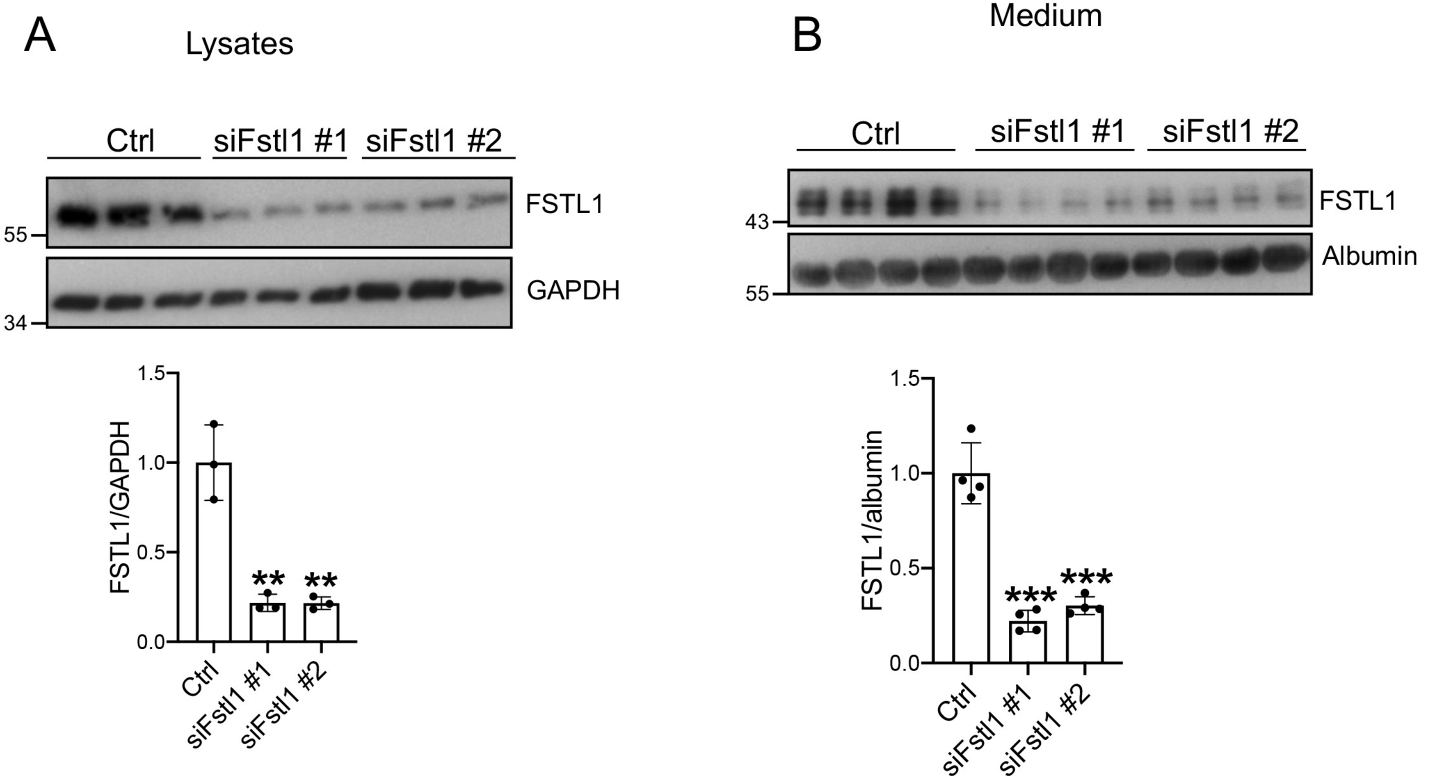

Fig. S14. Effects of rat Fstl1 siRNAs on FSTL1 expression in NRK49F cells. Cells were transfected with Scramble (Ctrl) or Fstl1 siRNAs (siFstl1, 110 nM). 24 h after transfection, cells were serum starved for 16 h before cells were harvested for real-time PCR analysis for Fstl1 mRNA levels (A) or for Western blotting for FSTL1 protein (B). Quantitative analysis of FSTL1 protein levels was performed by densitometry (C). n = 3 for panel A. $^{**}P < 0.01$; $^{***}P < 0.001$.

Fig. S15

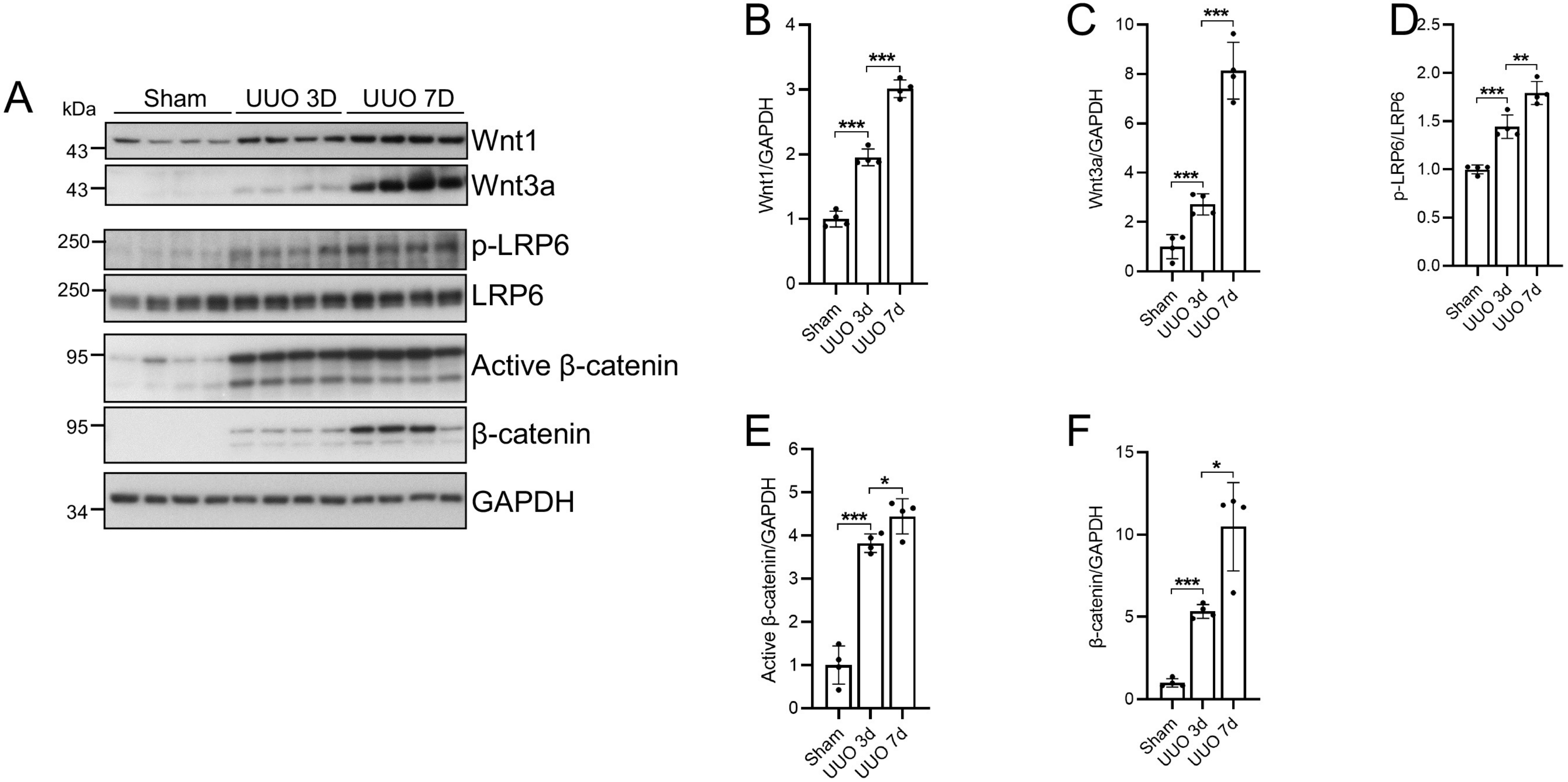

Fig. S15. The canonical Wnt/β-catenin pathway in the kidneys of mice subjected to UUO. 8-week-old male mice were subjected to UUO on the left ureters. Mice were sacrificed 3 and 7 days after UUO surgery. kidneys were collected and analyzed by Western blotting for Wnt1, Wnt3a, p-LRP6, LRP6, active β-catenin and β-catenin (A). Quantitative analysis was performed by densitometry, and levels of Wnt1 relative to GAPDH (B), Wnt3a relative to GAPDH (C), p-LRP6 relative to LRP6 (D), active β-catenin relative to GAPDH (E), and β-catenin relative to GAPDH (F) are presented. $*P<0.05$; $**P<0.01$; $***P<0.001$.

Fig. S16

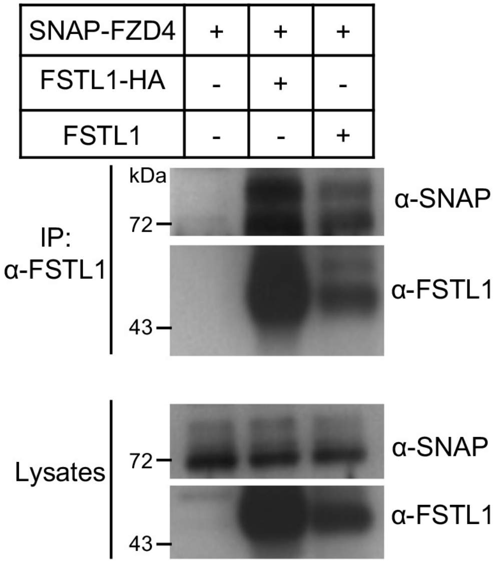

Fig. S16. FSTL1 interacts with SNAP-FZD4. HEK293T cells were transfected with SNAP-FZD4 (200 ng/ml) in the absence or presence of FSTL1-HA (100 ng/ml) or FSTL1 plasmid (200 ng/ml). Cell lysates were immunoprecipitated with anti-FSTL1, followed by Western blotting with anti-FSTL1 or anti-SNAP antibody on the precipitates or lysates as indicated. IP, immunoprecipitation.

Fig. S17

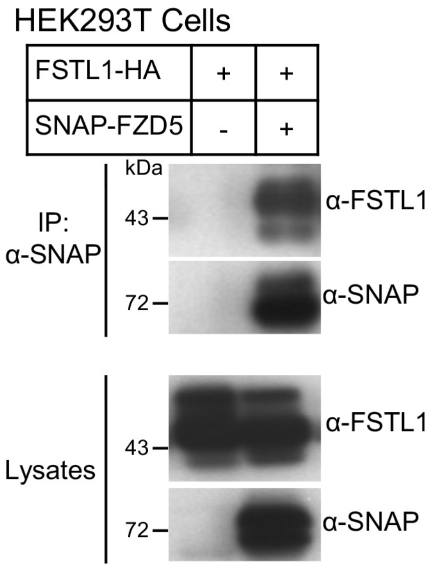

Fig. S17. FSTL1 interacts with FZD5. HEK293T cells were transfected with FSTL1-HA (100 ng/ml) in the absence or presence of SNAP-FZD5 plasmid (100 ng/ml). Cell lysates were immunoprecipitated with anti-SNAP, followed by Western blotting with anti-FSTL1 or anti-SNAP antibody on the precipitates or lysates as indicated. IP, immunoprecipitation.

Fig. S18

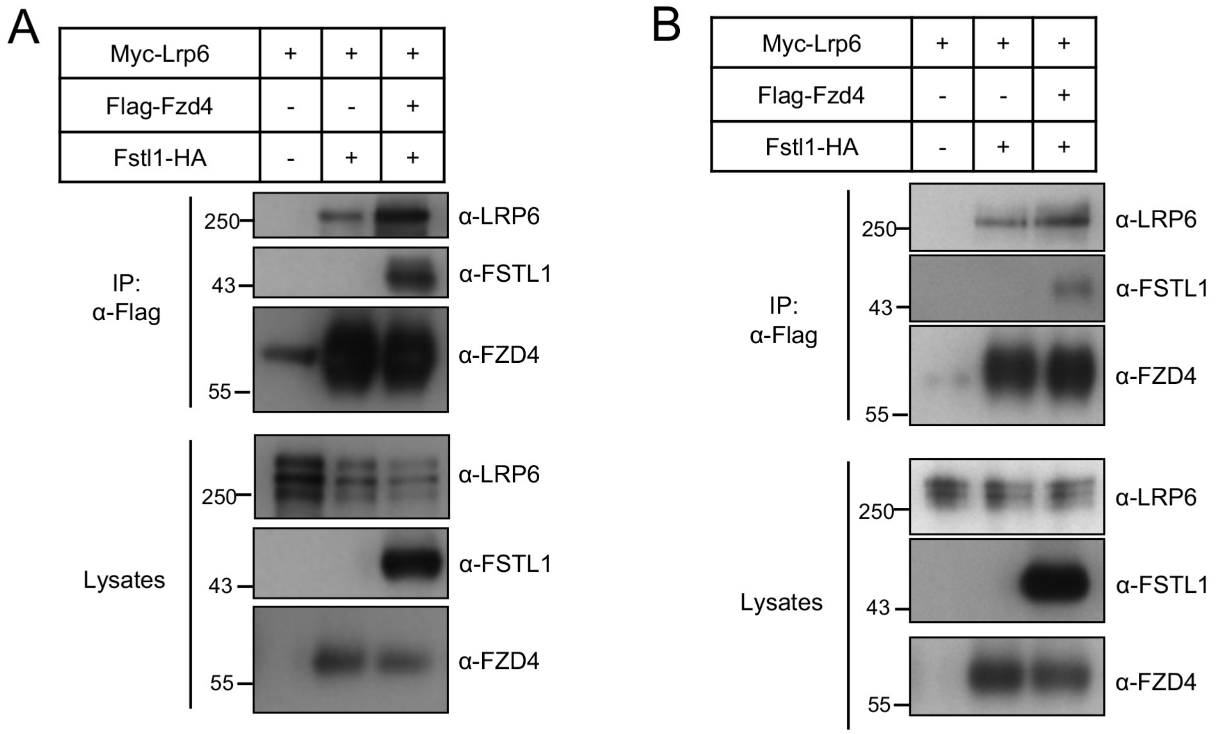

Fig. S18. Interaction of FZD4 and LRP6 in the presence of FSTL1. HEK293T cells were transfected with LRP6 plasmid in the absence or presence of FZD4 or FSTL1-HA plasmid. Cell lysates were immunoprecipitated with anti-Flag antibody. Western blotting was performed on whole lysates and precipitates as indicated. (A) and (B): two separate experiments.

Fig. S19

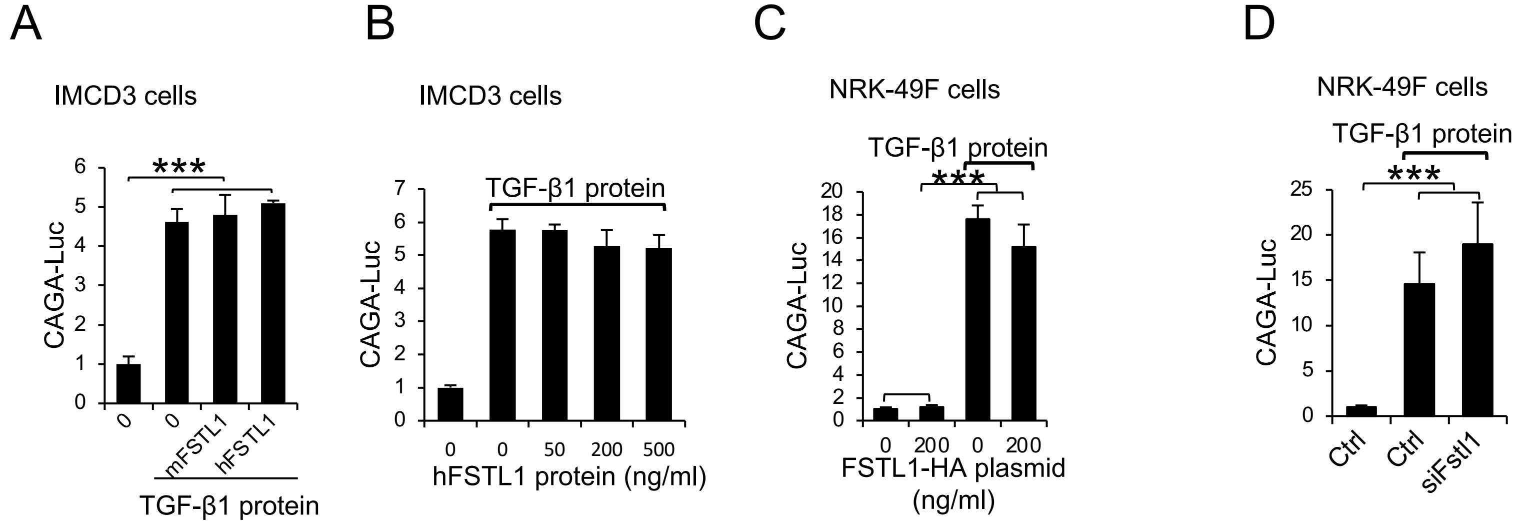

Fig. S19. Effects of FSTL1 on TGF-β signaling. (A) Neither mFSTL1 nor hFSTL1 proteins altered TGF-β1 mediated CAGA luciferase reporter activity in IMCD3 cells. Cells were transfected with CAGA luciferase reporter plasmid and treated with and without TGF-β1 (10 ng/ml) in the absence or presence of mFSTL1 or hFSTL1 (200 ng/ml). (B) Effects of increasing doses of hFSTL1 on TGF- β1 signaling in IMCD3 cells. Cells were transfected with CAGA luciferase reporter plasmid and treated with increasing doses of hFSLT1 (0-500 ng/ml), in the absence or presence of TGF- β1. (C) NRK-49F cells were co-transfected with CAGA luciferase reporter, in the absence of presence of FSTL1-HA plasmid and treated with and without TGF-β1 (20 ng/ml). (D) NRK-49F cells were co-transfected with CAGA luciferase reporter, in the presence of scrambled siRNA (Ctrl) or Fstl1 siRNAs and treated with and without TGF-β1 (20 ng/ml). ****P*<0.001.

Fig. S20

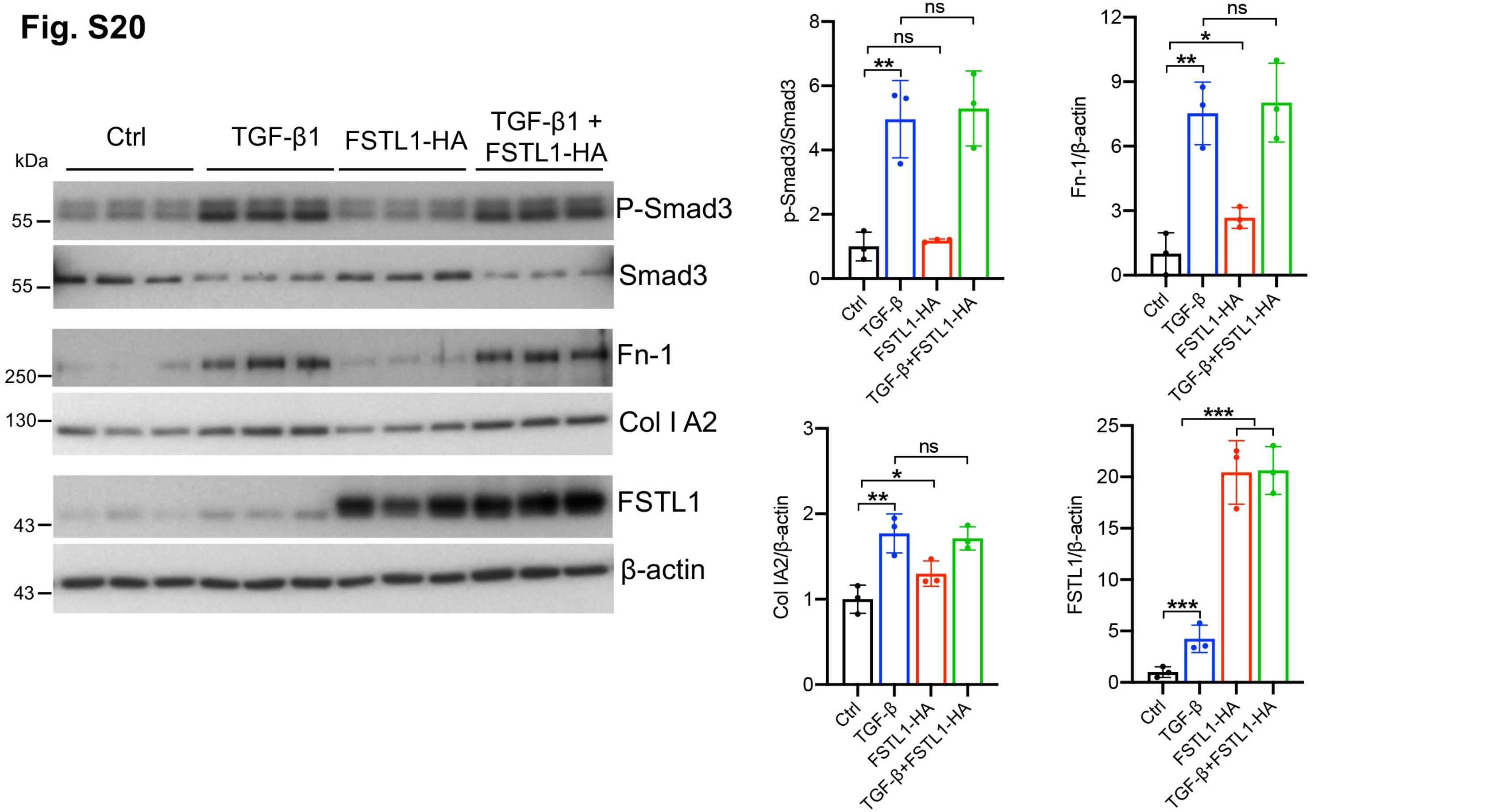

Fig. S20. Effects of FSTL1 on TGF-β signaling and activities in NRK-49F cells. Cells were transfected with and without FSTL1-HA plasmid (200 ng/ml). 24 h after transfection, cells were treated with and without TGF-β (20 ng/ml) in serum free medium for 24 h before cells were harvested for Western blotting for P-Smad3, Smad3, fibronectin (Fn-1), collagen 1A2 (Col1A2), and FSTL1. Levels of P-Smad3 relative to Smad3, and levels of Fn-1 and Col 1A2 relative to β-actin were also presented. *P<0.05; **P<0.01; ***P<0.001.
